# Saline and alkaline stresses alter soil properties and composition and structure of gene-based nitrifier and denitrifier communities in a calcareous desert soil

**DOI:** 10.1186/s12866-021-02313-z

**Published:** 2021-09-15

**Authors:** Jiaxin Guo, Yongxue Zhou, Huijuan Guo, Wei Min

**Affiliations:** Department of Resources and Environmental Science, Agriculture College, Shihezi University, Box #425, Shihezi, Xinjiang 832003 People’s Republic of China

**Keywords:** Chloride stress, Sulfate stress, Alkaline stresses, Bacterial community diversity, High-throughput sequencing, Potential nitrification rate

## Abstract

**Background:**

Saline and alkaline stresses damages the health of soil systems. Meanwhile, little is known about how saline or alkaline stress affects soil nitrifier and denitrifier communities. Therefore, we compared the responses of gene-based nitrifier and denitrifier communities to chloride (CS), sulfate (SS), and alkaline (AS) stresses with those in a no-stress control (CK) in pots with a calcareous desert soil.

**Results:**

Compared with CK, saline and alkaline stress decreased potential nitrification rate (PNR) and NO_3_-N; increased pH, salinity, water content, and NH_4_-N; and decreased copy numbers of *amoA*-AOA and *amoA*-AOB genes but increased those of denitrifier *nirS* and *nosZ* genes. Copies of *nirK* increased in SS and AS but decreased in CS. There were more copies of *amoA*-AOB than of *amoA*-AOA and of *nirS* than of *nirK* or *nosZ*. Compared with CK, SS and AS decreased operational taxonomic units (OTUs) of *amoA*-AOB but increased those of *nirS* and *nosZ*, whereas CS decreased *nirK* OTUs but increased those of *nosZ*. The numbers of OTUs and *amoA*-AOB genes were greater than those of *amoA*-AOA. There were positive linear relations between PNR and *amoA*-AOA and *amoA*-AOB copies. Compared with CK, the Chao 1 index of *amoA*-AOA and *amoA*-AOB decreased in AS, that of *nirK* increased in CS and SS, but that of *nirS* and *nosZ* increased in all treatments. The Shannon index of *amoA*-AOB decreased but that of *nirS* increased in CS and SS, whereas the index of *nirK* decreased in all treatments. Saline and alkaline stress greatly affected the structure of nitrifier and denitrifier communities and decreased potential biomarkers of *nirS*-type; however, AS increased those of *nirK*- and *nosZ*-type, and SS decreased those of *nosZ*-type. Soil water content, pH, and salinity were important in shaping *amoA*-AOA and denitrifier communities, whereas soil water and pH were important to *amoA*-AOB communities.

**Conclusion:**

These results indicate that the nitrifier and denitrifier communities respond to saline and alkaline stresses conditions. Communities of *amoA*-AOA and *amoA*-AOB contribute to nitrification in alluvial gray desert soil, and those of *nirS* are more important in denitrification than those of *nirK* or *nosZ*.

## Background

Salt stress is a primary threat to environmental resources and human health and also decreases crop yields and restricts the use of agricultural land [1]. Estimates suggest there are 1128 Mha of salt-affected land worldwide [2], accounting for more than 20% of total cultivated and 33% of irrigated agricultural lands [3]. In China, there are approximately 8.11 × 10^7^ ha of saline or alkaline soils, accounting for 8 to 9% of the total land area [4]. In general, salt-affected soils are either saline or alkaline. Alkaline salt stress and neutral salt stress are different and therefore should be correctly distinguished as alkaline stress and saline stress, respectively [5]. The effects of saline stress (NaCl or Na_2_SO_4_) are generally osmotic and ionic, whereas alkaline stress (NaHCO_3_ or Na_2_CO_3_) results from higher pH. Saline or alkaline stress adversely affects soil physicochemical properties, soil microbiological processes, and plant growth [6, 7].

Nitrification is the oxidation of ammonium (NH_4_^+^) to nitrite (NO_2_^−^) and then to nitrate (NO_3_^−^). The nitrification process primarily involves ammonia-oxidizing bacteria (AOB) or archaea (AOA) and nitrite-oxidizing bacteria; ammonia oxidation is the rate-limiting step in nitrification and is driven by AOA and AOB. Denitrification is the stepwise reduction of NO_3_^−^ and NO_2_^−^ to the gases nitric oxide (NO), nitrous oxide (N_2_O), and nitrogen (N_2_) under the catalysis of enzymes [8]. The reduction of NO_2_^−^ to NO is the rate-limiting step in denitrification. Nitrification and denitrification may occur simultaneously in different microsites of the same soil, and are affected by changes in salinity, pH, mineral N, soil water content (SWC), and temperature [9–13]. Soil salinity and pH affects nitrification and denitrification, primarily by inhibiting the activity of nitrifying and denitrifying bacteria. However, the response of N microbes to alkalinity or salinity stress is indeterminate.

Both AOA and AOB have *amoA* genes that encode ammonia monooxygenase to oxidize NH_4_-N. Ammonia oxidation is dominated by AOA in acidic environment or low nutrient content environment but by AOB in alkaline soils, because of the low survival of AOB at low pH [14–17]. Shi et al. found that AOB copies were positively correlated with soil water content, NH_4_-N, and NO_3_-N and negatively correlated to soil pH, indicating that AOB was mainly affected by soil water content, pH, NH_4_-N, and NO_3_-N [18]. In addition, previous studies found that copies of the *amoA* gene of AOB and AOA are negatively correlated with soil salinity [19, 20]. However, according to Wang and Gu, high soil salinity can promote the growth of AOB and AOA [21], and Mosier and Francis found that copies of *amoA* of AOB increase with an increase in soil salinity [22]. Therefore, how soil salinity affects the relative contributions of AOB and AOA to nitrification remains debatable. There is also little information on how pH and salinity affect the distributions of AOA and AOB in saline or alkaline soils.

The genes *nirK*, *nirS*, and *nosZ* are frequently used as functional markers to analyze denitrifier communities [23]. Soil salinity inhibits nitrification and denitrification rates [24, 25]. However, denitrifier communities respond differently to diverse environments. Salinity reduces copies of the denitrifier genes *nirK*, *nirS*, and *nosZ* and alters denitrifier community structure [26–28]. By contrast, Franklin et al. and Li et al. found that gene copies of denitrifying bacteria increased along gradients of increasing salinity [29, 30]. Thus, the effects of salinity on the abundance of soil denitrifying bacteria also remain unclear. Additionally, a pH-dependent mechanism is involved in regulating soil microbial community composition and function [31]. For example, Bai et al. found that increases in pH in saline soil increase the activity of denitrifying microorganisms [32]. These studies demonstrate that, owing to the complexity of soil microbial communities, the effects of saline or alkaline stress on the abundance and structure of nitrifier and denitrifier communities are poorly understood.

Nitrogen (N) is an essential nutrient needed to improve crop yields, and many farmers apply excess N fertilizer to ensure maximum yield and profit [33]. Soil microbial communities have essential roles in nutrient cycling, and many of the microbiological processes involved in N cycling in terrestrial ecosystems are altered under saline or alkaline stress, which can affect plant productivity and production of atmospherically active gases. An understanding of the effects of saline and alkaline stresses on the abundance of nitrifiers and denitrifiers is important, because nitrification and denitrification rates determine soil inorganic N concentrations, nitrate leaching, and the production of N_2_O [20, 27, 34]. An increase in soil salinity may shift microbial community structure and increase the predominance of saline or alkaline adapted microorganisms. However, there are few reports focused on the effects of saline and alkaline stresses on composition and structure of gene-based nitrifier and denitrifier communities, and the results will help to guide the application of saline and alkaline soil.

In this study, the effects of saline and alkaline stresses on nitrifier and denitrifier abundance and community structure were determined. We hypothesized that (i) saline and alkaline stresses would have different effects on nitrifier and denitrifier abundance, but overall, an increase in soil salinity or alkalinity would decrease the abundance of both, and that (ii) saline and alkaline stresses would have different effects on nitrifier and denitrifier community structure, but with an increase in salinity or alkalinity, the predominance of saline or alkaline-adapted microorganisms would increase. The hypotheses were tested in a pot experiment with cotton plants and different types of soil salinity and alkalinity stresses. We assessed nitrifier and denitrifier abundance by quantitative polymerase chain reaction (q-PCR) and community structure by 16S rRNA gene sequencing. The information obtained in this study can provide a theoretical basis for the efficient use of N fertilizers and rational N management in saline or alkaline soils in arid areas.

## Results

### Cotton biomass

Cotton biomass decreased significantly under salt–alkali stress (Table 1). Compared with the CK, the biomass of the leaves, stem, and root was significantly lower by 47.55, 65.68, and 32.26%, respectively, in the CS treatment, by 46.85, 50.89, and 43.01%, respectively, in the SS treatment, and by 60.14, 57.40, and 31.18%, respectively, in the AS treatment. Overall, compared with the CK, total cotton biomass was decreased by 51.94, 47.74, and 53.18% in the CS, SS, and AS treatment, respectively.

**Table 1:** Component biomass and total biomass of cotton plants as affected by salt and alkali stresses in a calcareous desert soil

Treatments	Biomass (g/plant)			
	Leaves	Stems	roots	Total
CK	7.15 a	7.61 a	3.72 a	18.48 a
CS	3.75 b	2.61 d	2.52 b	8.88 c
SS	3.80 b	3.74 b	2.12 c	9.66 b
AS	2.85 c	3.24 c	2.56 b	8.65 c

CK, control treatment without salt or alkali stress; CS, NaCl stress treatment; SS, Na_2_SO_4_ stress treatment; and AS, Na_2_CO_3_ + NaHCO_3_ stress treatment. Different lowercase letters in the same column indicate significant differences among treatments (*P* < 0.05)

### Soil physicochemical properties and potential nitrification rate

**Fig. 1:**
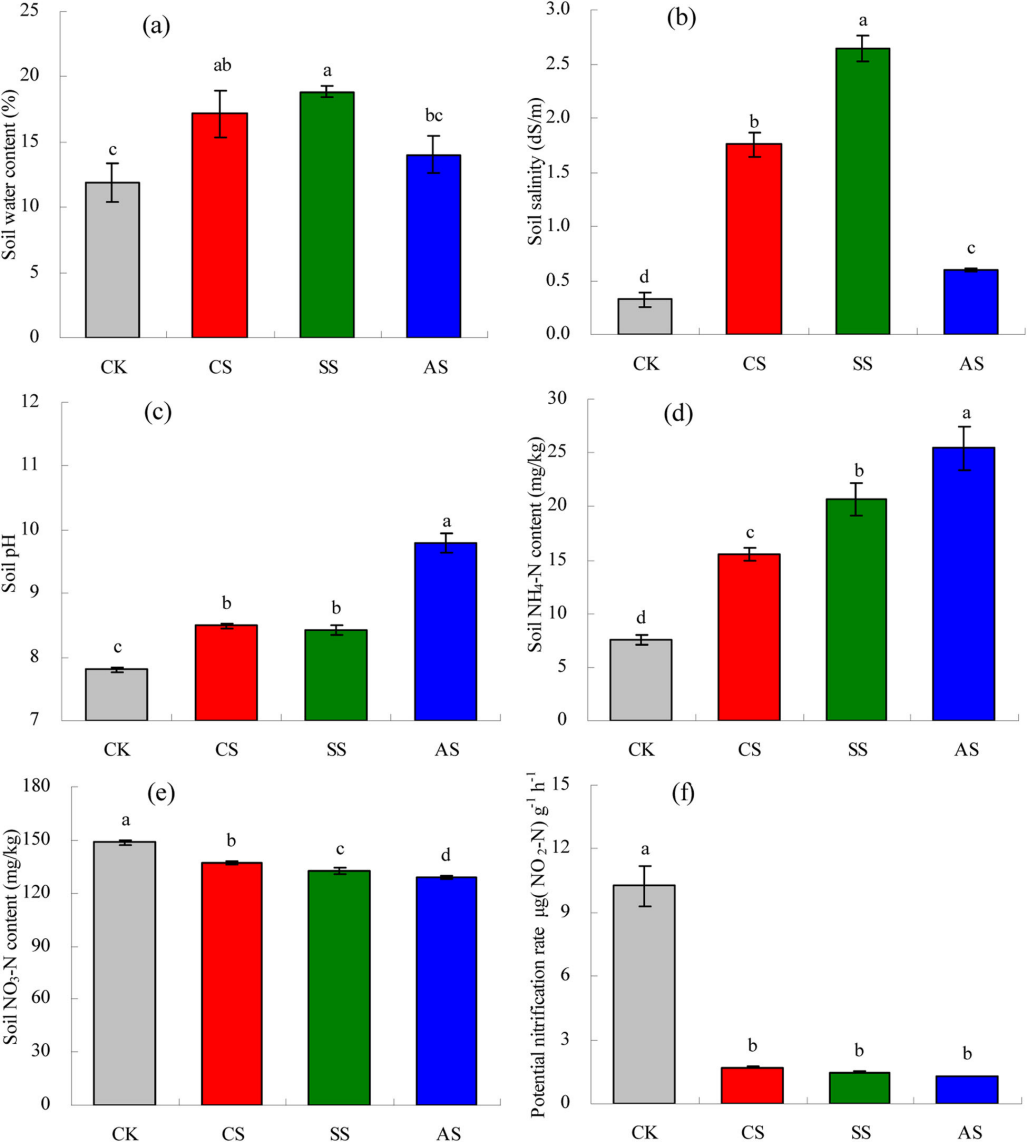
Effects of different types of salt and alkali stresses on soil properties and nitrification in a calcareous desert soil. **a** Soil water content (%), **b** salinity (dS m^−1^), **c** pH, **d** NH_4_-N (mg kg^−1^), **e** NO_3_-N (mg kg^−1^), and **f** potential nitrification rate (μg NO_2_-N g^−1^ h^−1^). Columns and error bars represent the mean ± standard error (*n* = 3), respectively. CK, control treatment without salt or alkali stress; CS, NaCl stress treatment; SS, Na_2_SO_4_ stress treatment; AS, Na_2_CO_3_ + NaHCO_3_ stress treatment

Saline and alkaline stresses significantly increased SWC (Fig. 1a) and salinity (Fig. 1b). Across all treatments, including CK, SWC was between 11.88 and 18.87%, and salinity was between 0.33 and 2.64 dS m^− 1^. Compared with CK, SWC was 44.59% higher in CS, 58.82% higher in SS, and 18.05% higher in AS, and salinity was 438.41% higher in CS 708.99% higher in SS, and 83.25% higher in AS. Compared with that in CK, Saline and alkaline stresses significantly increased soil pH (Fig. 1c). In CS and SS, the pH increased by 0.69 and 0.63 units, respectively, compared with that in CK. In AS, the pH was significantly higher than that in the other treatments and was approximately 2.00 units higher than that in CK. The NH_4_-N content increased significantly in CS, SS, and AS, compared with that in CK (Fig. 1d), increasing by 106.29% in CS, 173.54% in SS, and 236.74% in AS. By contrast, the NO_3_-N content decreased significantly in CS, SS, and AS, compared with that CK, decreasing by 7.68% in CS, 10.68% in SS, and 13.47% in AS (Fig. 1e). Similarly, the soil PNR decreased significantly in CS, SS, and AS, compared with that in CK (Fig. 1f), decreasing by 501% in CS, 608% in SS, and 697% in AS. There were no significant differences in PNR among CS, SS, and AS.

### *amoA-AOA, amoA-AOB, nirK, nirS,* and *nosZ* gene copy numbers

**Fig. 2:**
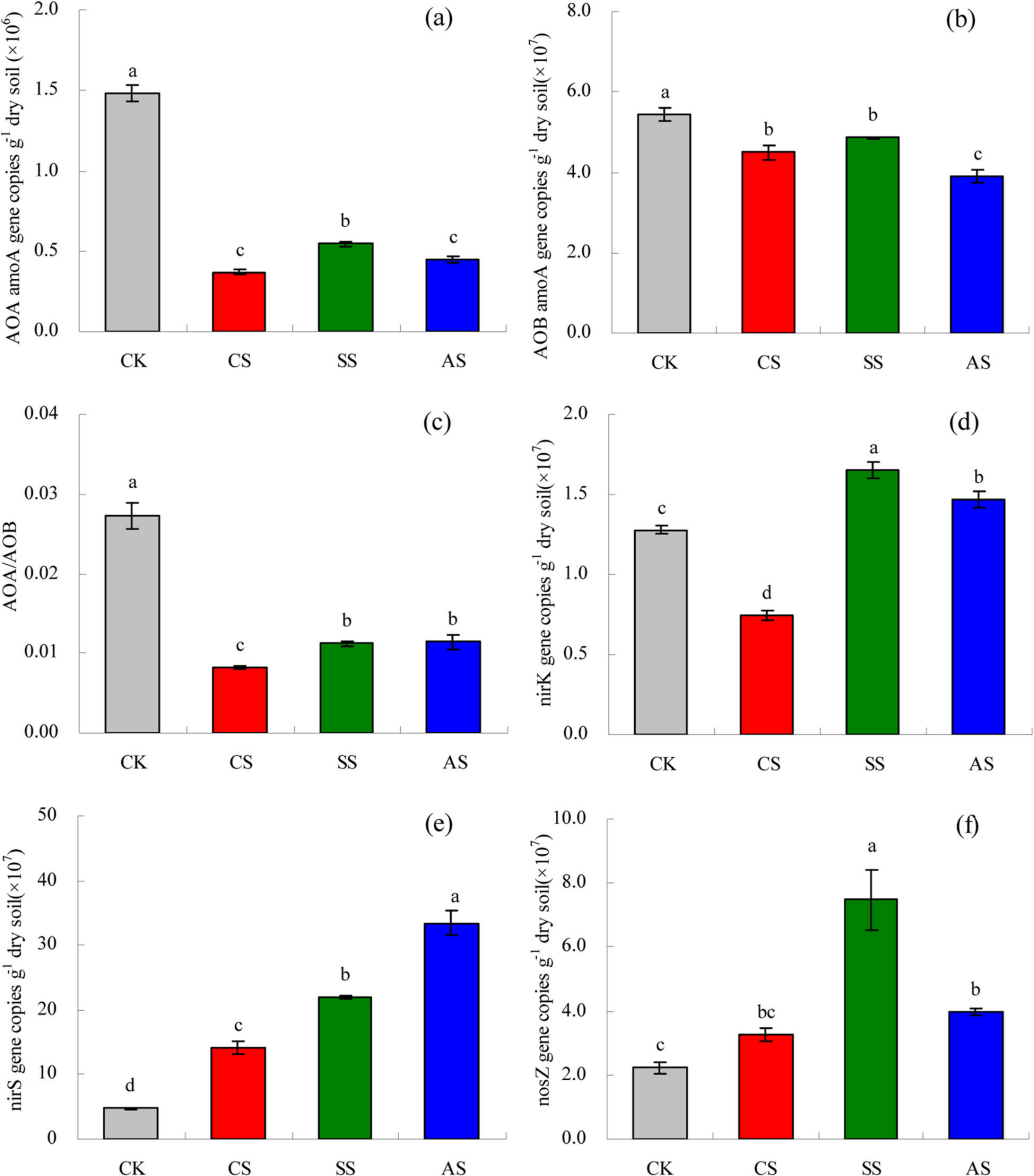
Effects of different types of salt and alkali stresses on gene copy numbers (no. g^− 1^ dry soil (× 10^6^ or 10^7^)) of different soil bacterial communities involved in nitrification and denitrification in a calcareous desert soil. **a** *amoA*-AOA, **b** *amoA*-AOB, **c** *amoA*-AOA/*amoA*-AOB ratio, **d** *nirK*, **e** *nirS*, and **f** *nosZ*.. Columns and error bars represent the mean ± standard error (*n* = 3), respectively. CK, control treatment without salt or alkali stress; CS, NaCl stress treatment; SS, Na_2_SO_4_ stress treatment; AS, Na_2_CO_3_ + NaHCO_3_ stress treatment

Saline and alkaline stresses significantly decreased *amoA*-AOA (Fig. 2a) and *amoA*-AOB (Fig. 2b) gene copy numbers. Across all treatments, including CK, gene copy numbers of *amoA*-AOA were between 0.44 × 10^6^ and 1.48 × 10^6^ per g dry soil and those of *amoA*-AOB were between 3.89 × 10^7^ and 5.45 × 10^7^ per g dry soil. Thus, the gene copy numbers of *amoA*-AOB were higher than those of *amoA*-AOA. The *amoA*-AOA and *amoA*-AOB gene copies in CS, SS, and AS were significantly lower than those in CK. For *amoA*-AOA gene copies, the number was not significantly different between CS and AS. For *amoA*-AOB gene copies, the number was not significantly different between CS and SS. Compared with CK, *amoA*-AOA gene copies were 75.18% lower in CS, 63.34% lower in SS, and70.08% lower in AS. Compared with CK, *amoA*-AOB gene copies were 17.48% lower in CS, 10.87% lower in SS, and 28.46% lower in AS. The *amoA*-AOA/*amoA*-AOB ratio in CS, SS, and AS was significantly lower than that in CK (Fig. 2c), but there was no significant difference between SS and AS.

Figure 2d, e, and f show the copy numbers of the denitrification genes *nirK*, *nirS*, and *nosZ*, respectively. Across all treatments, including CK, the numbers of *nirK* were between 0.74 × 10^7^ and 1.65 × 10^7^ per g dry soil, those of *nirS* between 4.69 × 10^7^ and 33.34 × 10^8^ per g dry soil, and those of *nosZ* between 2.23 × 10^7^ and 7.49 × 10^7^ per g dry soil. Compared with CK, the *nirK* copies decreased in CS by 41.54% but increased significantly by 28.94% in SS and by 14.71% in AS. Saline and alkaline stresses significantly increased *nirS* and *nosZ* gene copy numbers, compared with those in CK. The *nirS* copy numbers increased by 201.51% in CS, 368.18% in SS, and 612.57% in AS. The *nosZ* copy numbers increased by 46.44% in CS, 235.86% in SS, and 78.28% in AS.

### Relations between potential nitrification rate and abundances of *amoA-AOA* and *amoA-AOB*

**Fig. 3:**
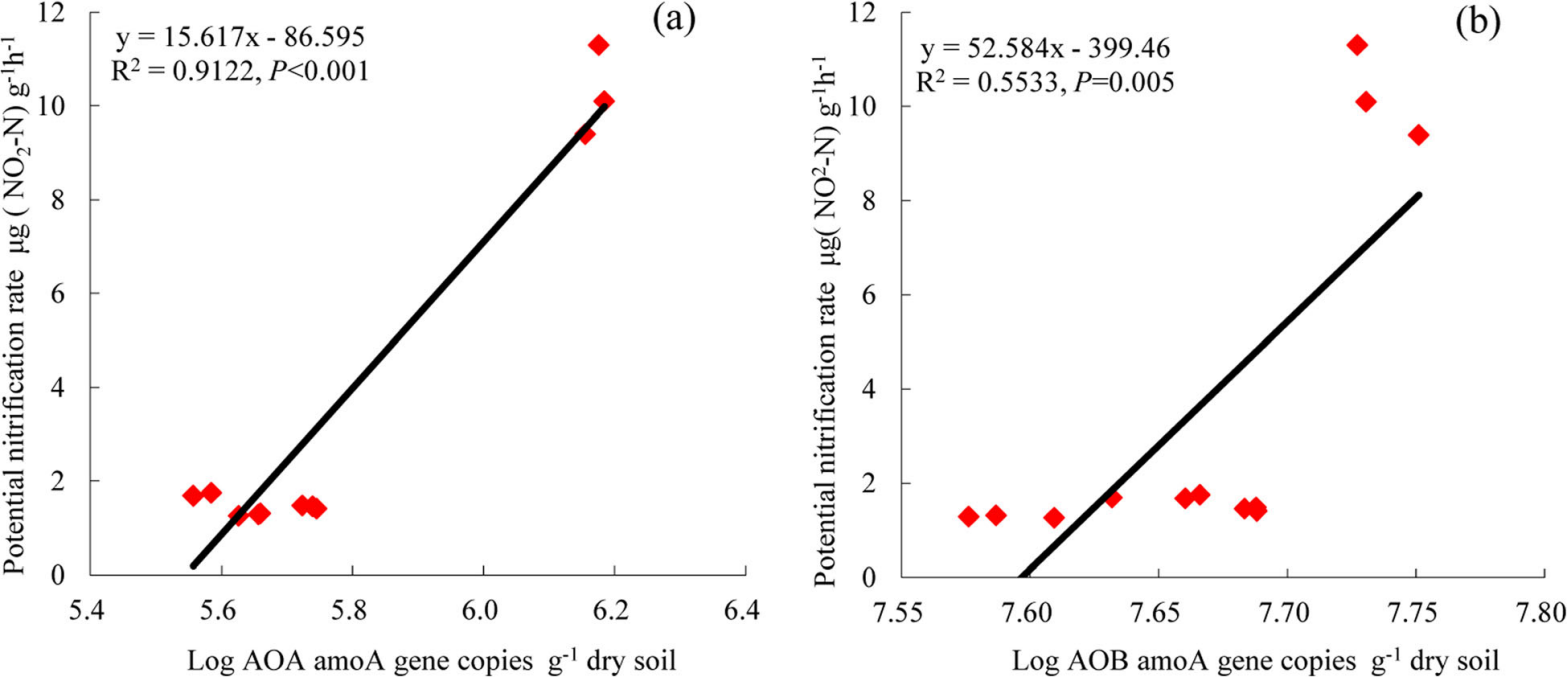
Regressions of the relations between potential nitrification rate (μg NO_2_-N g^− 1^ h^− 1^) and gene copy numbers (log no. g^− 1^ dry soil) of **a** *amoA*-AOA communities and **b** *amoA*-AOB communities in a calcareous desert soil

Regression analysis showed soil PNR was significantly positively related to the gene copy numbers of both *amoA*-AOA (*R*^2^ = 0.9122, *P* < 0.001; Fig. 3a) and *amoA*-AOB (*R*^2^ = 0.5533, *P* = 0.005; Fig. 3b). Thus, the PNR was highly linearly related to the abundances of *amoA*-AOA and *amoA*-AOB.

### Correlations between soil properties and potential nitrification rate and abundances of *amoA-AOA, amoA-AOB, nirK, nirS,* and *nosZ genes*

Looking first at the relationships between the soil’s physicochemical properties and its PNR and nitrifier communities gene abundances (Table 2). The soil PNR was significantly negatively correlated with SWC, EC_1:5_, pH, and NH_4_-N content, the abundance of *amoA*-AOA was significantly negatively correlated with SWC, pH, and NH_4_-N content, and the abundance of *amoA*-AOB was significant negatively correlated with pH and NH_4_-N content. However, soil PNR and the abundance of *amoA*-AOA and *amoA*-AOB were significantly positively correlated with soil NO_3_-N content.

**Table 2:** Pearson coefficients of correlation between soil properties and copy numbers of the genes *amoA*-AOA, *amoA*-AOB, *nirK*, *nirS*, and *nosZ* in a calcareous desert soil

Item	PNR	*amoA*-AOA	*amoA*-AOB	*nirK*	*nirS*	*nosZ*
SWC	−0.675 ^a^	−0.658 ^a^	−0.179	0.026	0.250	0.712 ^b^
EC_1:5_	−0.604 ^a^	−0.563	−0.015	0.094	0.142	0.813 ^b^
pH	−0.673 ^a^	−0.662 ^a^	−0.918 ^b^	0.220	0.935 ^b^	0.136
NH_4_-N	−0.857 ^b^	−0.798 ^b^	−0.848 ^b^	0.409	0.965 ^b^	0.571
NO_3_-N	0.914 ^b^	0.869 ^b^	0.856 ^b^	−0.319	−0.942 ^b^	−0.587 ^a^

*EC*_*1:5*_ electrical conductivity, *SWC* soil water content, *PNR* potential nitrification rate

^a^ and ^b^ indicate significant correlations at the 0.05 and 0.01 levels (two-tailed), respectively

Looking at the relationship between the soil’s physicochemical properties and denitrifier communities gene abundances, The abundance of *nirS* was significantly positively correlated with soil pH and soil NH_4_-N content, and the abundance of *nosZ* was significantly positively correlated with SWC and EC_1:5_, but the abundance of *nirS* and *nosZ* was significantly negatively correlated with soil NO_3_-N content. In addition, the abundance of *nirK* was not significantly correlated with any soil property.

### Venn diagrams of the operational taxonomic units of *amoA-AOA, amoA-AOB, nirK, nirS,* and *nosZ genes*

The sequence coverage of *amoA*-AOA, *amoA*-AOB, *nirK*, *nirS*, and *nosZ* genes was greater than 99% in all samples, indicating that the depth reasonably represented the actual communities (Table 3). Saline and alkaline stresses significantly affected the number of OTUs and sequences. For *amoA*-AOB, the number of OTUs decreased significantly in SS and AS, compared with that in CK. For *nirK*, the number of OTUs decreased significantly in CS and SS, compared with that in CK. However, for *nirS*, the number of OTUs increased significantly in SS and AS, compared with that in CK and for *nosZ*, the number increased significantly in CS, SS, and AS. In addition, For *amoA*-AOB, the number of sequences decreased significantly in CS, compared with that in CK, however, the number of sequences increased significantly in AS. For *nirK*, the number of sequences increased significantly in CS, compared with that in CK. For *nosZ*, the number of sequences increased significantly in CS, SS, and AS, compared with that in CK.

**Table 3:** Number of operational taxonomic units (OTUs), sequences, and coverages of the genes used to identify nitrifier and denitrifier communities under salt and alkali stresses in a calcareous desert soil

Treatments	OTUs					Sequences					Coverages				
	*amoA*-AOA	*amoA-*AOB	*nirK*	*nirS*	*nosZ*	*amoA-*AOA	*amoA-*AOB	*nirK*	*nirS*	*nosZ*	*amoA-*AOA	*amoA-*AOB	*nirK*	*nirS*	*nosZ*
CK	31 a	324 a	377 a	285 b	178 c	37,110 a	42,345 b	51,095 b	29,293 a	33,242 b	0.9998	0.9954	0.9980	0.9979	0.9993
CS	34 a	312 a	270 b	310 b	232 b	40,672 a	35,221 c	56,195 a	28,503 a	40,047 a	0.9998	0.9948	0.9983	0.9975	0.9986
SS	36 a	271 b	284 b	353 a	314 a	39,192 a	48,961 a	52,321 b	29,144 a	39,112 a	0.9998	0.9953	0.9982	0.9973	0.9979
AS	33 a	165 c	336 a	348 a	295 a	40,583 a	40,662 b	50,138 b	28,254 a	39,385 a	0.9999	0.9978	0.9980	0.9973	0.9985

CK, control treatment without salt or alkali stress; CS, NaCl stress treatment; SS, Na_2_SO_4_ stress treatment; and AS, Na_2_CO_3_ + NaHCO_3_ stress treatment. Different lowercase letters in the same column indicate significant differences among treatments (*P* < 0.05)

**Fig. 4:**
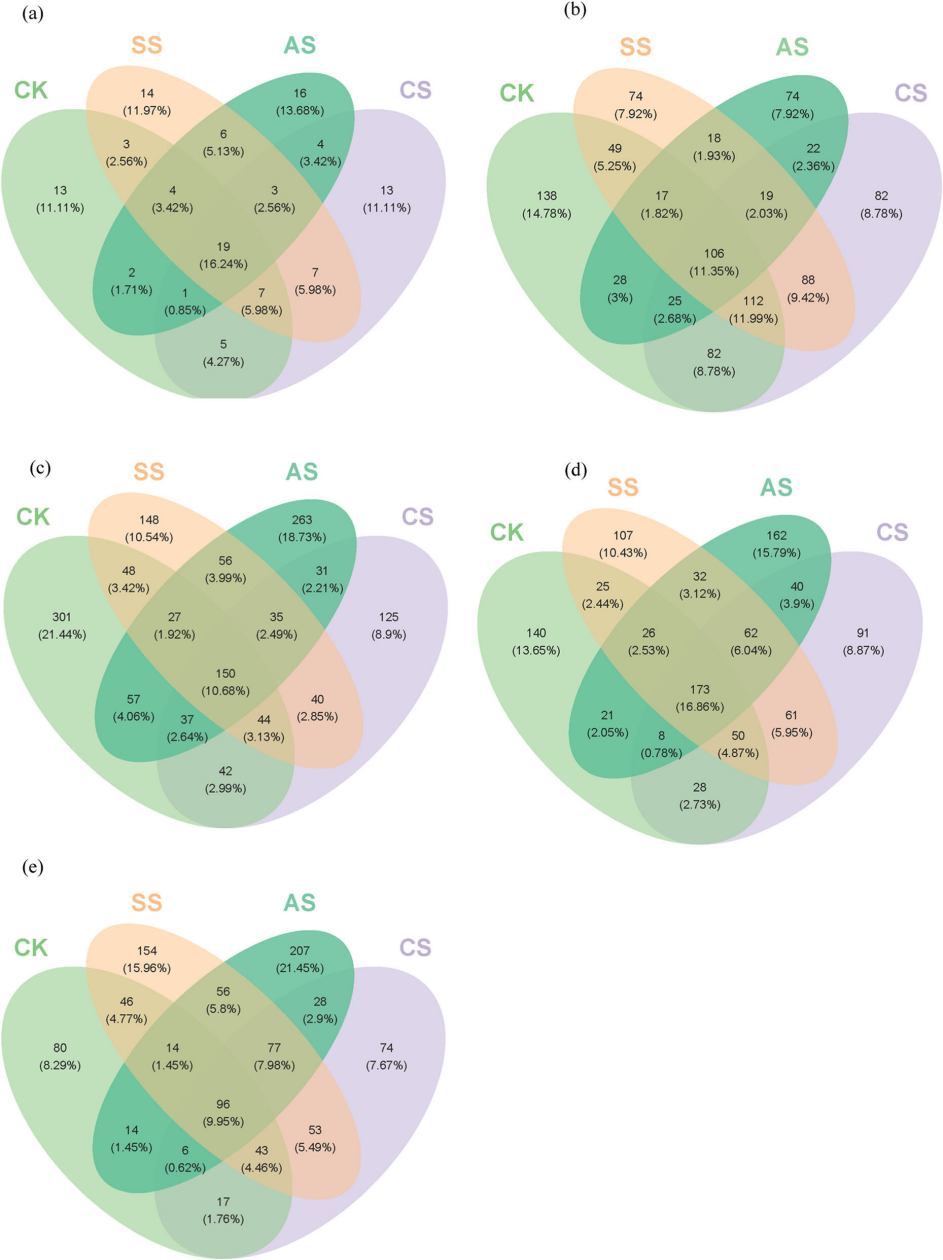
Venn diagrams based on operational taxonomic units of different communities under different salt and alkali stresses in a calcareous desert soil. The communities were **a** *amoA*-AOA, **b** *amoA*-AOB, **c** *nirK*, **d** *nirS*, and **e** *nosZ*. CK, control treatment without salt or alkali stress; CS, NaCl stress treatment; SS, Na_2_SO_4_ stress treatment; AS, Na_2_CO_3_ + NaHCO_3_ stress treatment

Venn diagrams were used to compare the shared and unique OTUs among *amoA*-AOA, *amoA*-AOB, *nirK*, *nirS*, and *nosZ* communities (Fig. 4). One hundred and seventeen *amoA*-AOA-related OTUs were identified in all treatments, and 19 were shared among the four treatments (16.24% of the total) (Fig. 4a). Nine hundred and thirty-four *amoA*-AOB-related OTUs were identified in all treatments, and 106 were shared among the four treatments (11.35% of the total) (Fig. 4b). The number of *amoA*-AOB species was significantly greater than that of *amoA*-AOA species. Furthermore, the saline and alkaline stresses had greater influence on the number of *amoA*-AOB-related OTUs than on the number of *amoA*-AOA-related ones.

As shown in Fig. 4c, 1,404 *nirK*-related OTUs were identified in all treatments, with 150 shared among the four treatments (10.68% of the total). As shown in Fig. 4d, 1,026 *nirS*-related OTUs were identified in all treatments, with 173 shared among the four treatments (16.86% of the total). As shown in Fig. 4e, 965 *nosZ*-related OTUs were identified in all treatments, with 96 shared among the four treatments (9.95% of the total). The numbers of *nirK* and *nirS* species were significantly than that of *nosZ* species. Furthermore, the saline and alkaline stresses had greater influence on the numbers of *nirK*- and *nirS*-related OTUs than on the numbers of *nosZ*-related ones.

### α-Diversity of *amoA-AOA, amoA-AOB, nirK-, nirS-,* and *nosZ-type* denitrifier communities

Table 4 shows the Chao1 and Shannon diversity indices of *amoA*-AOA, *amoA*-AOB, *nirK*-, *nirS*-, and *nosZ*-type denitrifier communities. Compared with CK, CS and SS had no effect on the Chao 1 index of *amoA*-AOA and *amoA*-AOB communities; however, AS significantly decreased the Chao 1 index of *amoA*-AOA and *amoA*-AOB communities. Compared with CK, CS significantly decreased the Shannon index of *amoA*-AOA and *amoA*-AOB communities. In SS the Shannon index of the *amoA*-AOB community decreased significantly.

**Table 4:** Diversity indices of *amoA*-AOA, *amoA*-AOB, *nirK*, *nirS*, and *nosZ* communities under different salt and alkali stresses in a calcareous desert soil

	Chao1					Shannon				
	*amoA*-AOA	*amoA*-AOB	*nirK*	*nirS*	*nosZ*	*amoA*-AOA	*amoA*-AOB	*nirK*	*nirS*	*nosZ*
CK	43.51 a	486.03 a	425.56 a	324.68 b	194.53 c	2.07 ab	2.60 a	4.80 a	4.40 b	0.67 a
CS	43.90 a	543.02 a	360.05 b	372.77 a	263.40 b	1.80 b	1.90 b	4.04 c	5.09 a	0.62 a
SS	41.62 a	516.82 a	362.20 b	402.67 a	367.40 a	2.53 a	1.82 b	3.93 c	5.19 a	0.69 a
AS	33.42 b	258.74 b	413.12 a	396.40 a	326.62 a	2.50 a	2.61 a	4.31 b	4.24 b	0.65 a

CK, control treatment without salt or alkali stress; CS, NaCl stress treatment; SS, Na_2_SO_4_ stress treatment; and AS, Na_2_CO_3_ + NaHCO_3_ stress treatment. Different lowercase letters in the same column indicate significant differences among treatments (*P* < 0.05)

Compared with CK, CS and SS significantly decreased the Chao1 index of the *nirK* community, whereas the same treatments significantly increased the index of the *nirS* and *nosZ* communities. In addition, the Shannon index of the *nirK* community decreased significantly in CS and SS, but in those same treatments, the index of the *nirS* community increased significantly. The Shannon index of the *nosZ*-type denitrifier community was not affected by any treatment.

### Nonmetric multidimensional scaling analysis

**Fig. 5:**
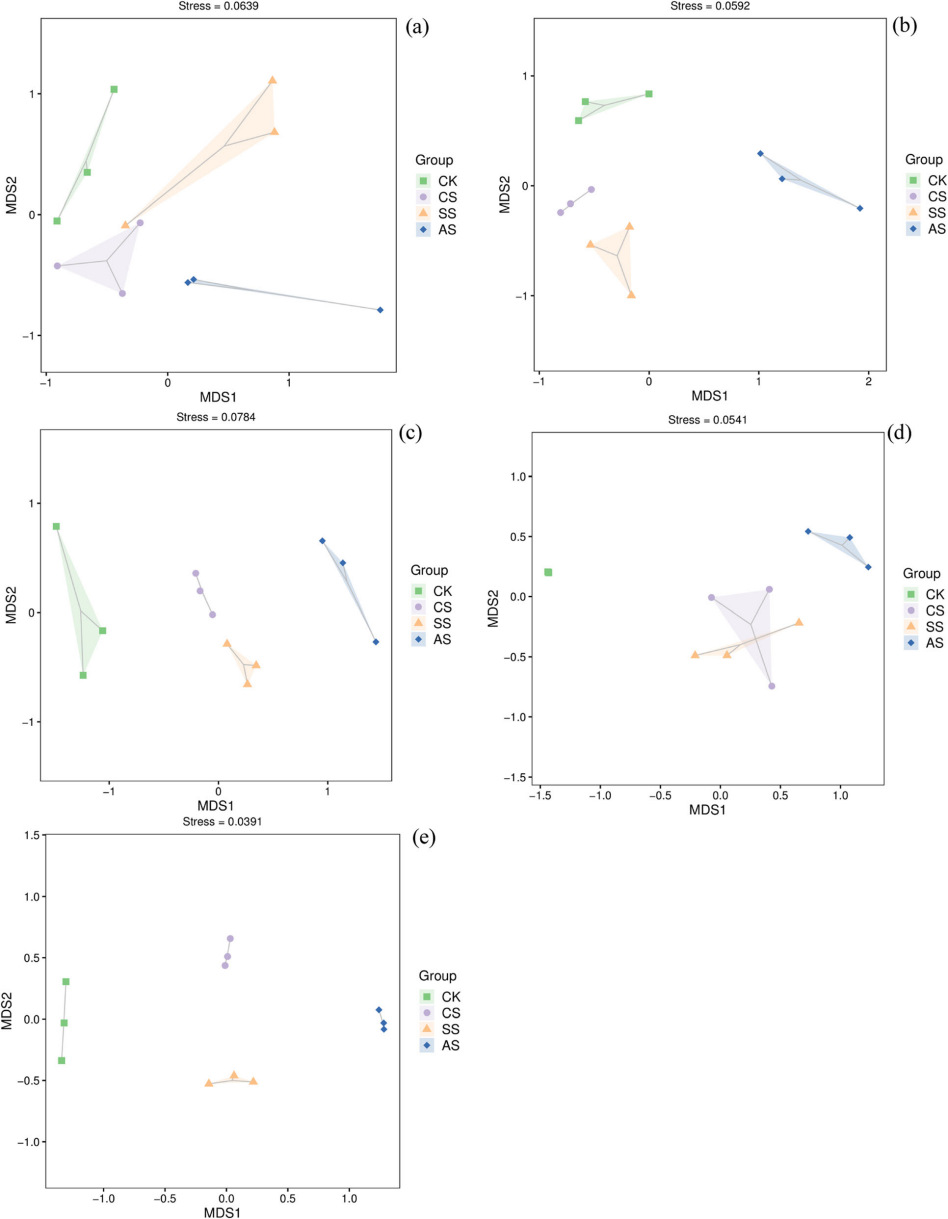
Non-metric multidimensional scaling (NMDS) analysis of different soil microbial communities involved in nitrification and denitrification under different salt and alkali stresses in a calcareous desert soil. The communities were **a** *amoA*-AOA, **b** *amoA*-AOB, **c** *nirK*, **d** *nirS*, **e** and *nosZ*. CK, control treatment without salt or alkali stress; CS, NaCl stress treatment; SS, Na_2_SO_4_ stress treatment; AS, Na_2_CO_3_ + NaHCO_3_ stress treatment

Nonmetric multidimensional scaling analysis was performed to compare the differences in structure of *amoA*-AOA, *amoA*-AOB, *nirK*, *nirS*, and *nosZ* communities among treatments (Fig. 5). The *amoA*-AOA, *amoA*-AOB, *nirK*, and *nosZ* communities were clustered into four groups (Fig. 5a, b, c, d). The structure of *nosZ* communities was not significant between CS and SS (Fig. 5e). However, the structure of the *nosZ* communities under saline and alkaline stresses was significantly different from that of those communities in CK. This result indicated that microbial community structure might be directly correlated with soil properties affected by saline and alkaline stresses.

### Composition of *amoA-AOA, amoA-AOB, nirK-, nirS-,* and *nosZ-type* denitrifier communities

**Fig. 6:**
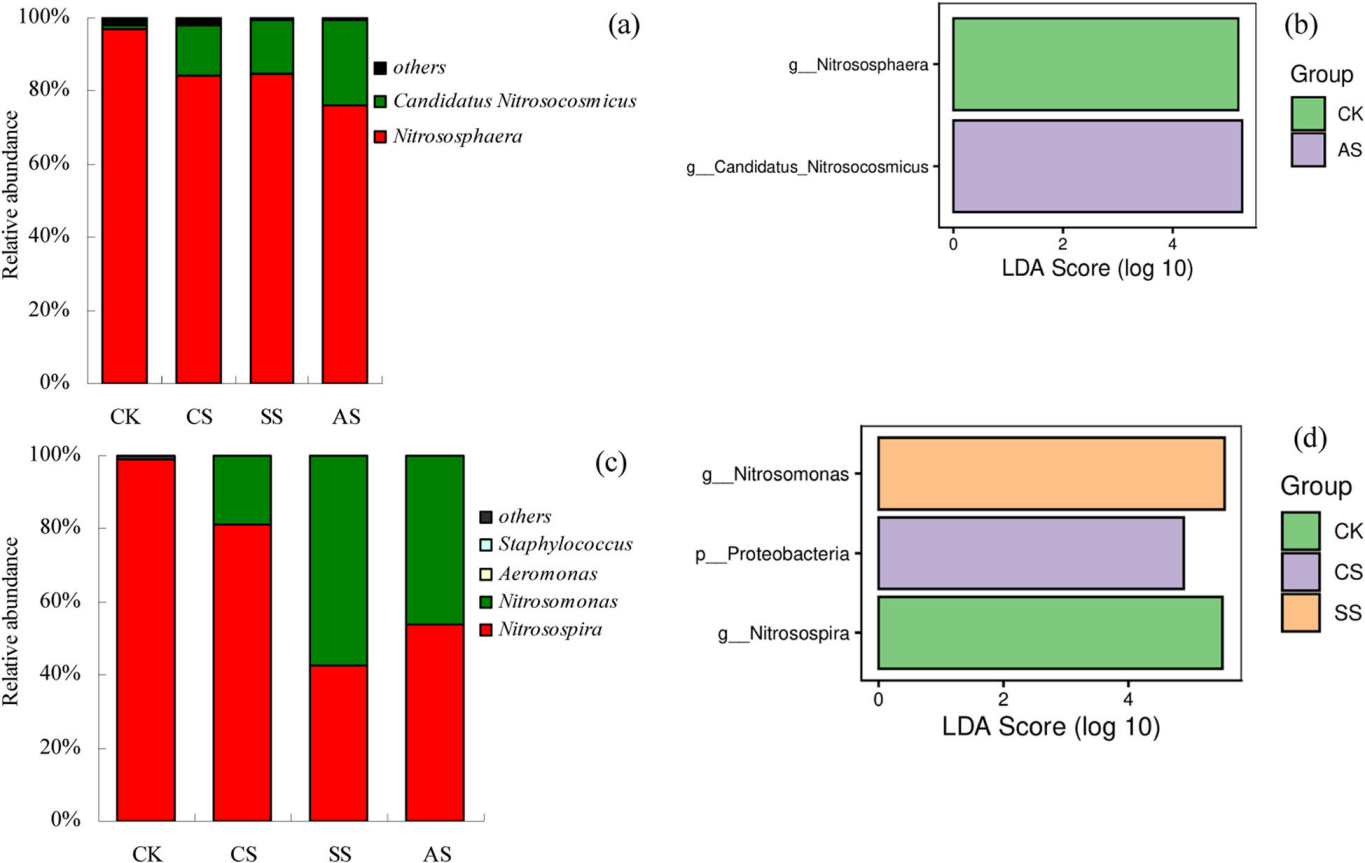
Genus-level composition (**a**, **c**) and linear discriminant analysis (LDA) effect size analysis (**b**, **d**) of bacterial communities based on nitrification functional genes under different types of salt and alkali stresses in a calcareous desert soil. **a** Relative abundance (%) of genera in *amoA*-AOA communities, **b** LDA scores of dominant genera in *amoA*-AOA communities, **c** relative abundance (%) of genera in *amoA*-AOB communities, and **d** LDA scores of dominant taxa in *amoA*-AOB communities. CK, control treatment without salt or alkali stress; CS, NaCl stress treatment; SS, Na_2_SO_4_ stress treatment; AS, Na_2_CO_3_ + NaHCO_3_ stress treatment

Saline and alkaline stresses significantly affected the genus-level composition of *amoA*-AOA and *amoA*-AOB communities (Fig. 6). In the *amoA*-AOA community, the two dominant genera were *Nitrososphaera* and *Candidatus Nitrosocosmicus* (Fig. 6a). In all treatments, *Nitrososphaera* had the highest relative abundance (76.08 to 96.94%). The relative abundance of *Nitrososphaera* was significantly higher in CK than that in CS, SS, and AS. However, the relative abundance of *Candidatus Nitrosocosmicus* was significantly higher in CS, SS, and AS than that in CK. *Nitrososphaera* (96.94%) was dominant in CK, and *Candidatus Nitrosocosmicus* (23.66%) was significantly enriched in AS (Fig. 6b).

In the *amoA*-AOB community, *Nitrosospira* and *Nitrosomonas* were the two dominant genera (Fig. 6c). Across all treatments, including CK, the relative abundance of *Nitrosospira* was between 42.41 and 99.04% and that of *Nitrosomonas* between 0.01 and 57.53%. The relative abundance of *Nitrosospira* was significantly higher in CK than that in CS, SS, and AS. However, the relative abundance of *Nitrosomonas* was significantly higher in CS, SS, and AS than that in CK. *Nitrosospira* (99.04%) was dominant in CK, and *Nitrosomonas* (57.53%) was significantly enriched in SS (Fig. 6d).

**Fig. 7:**
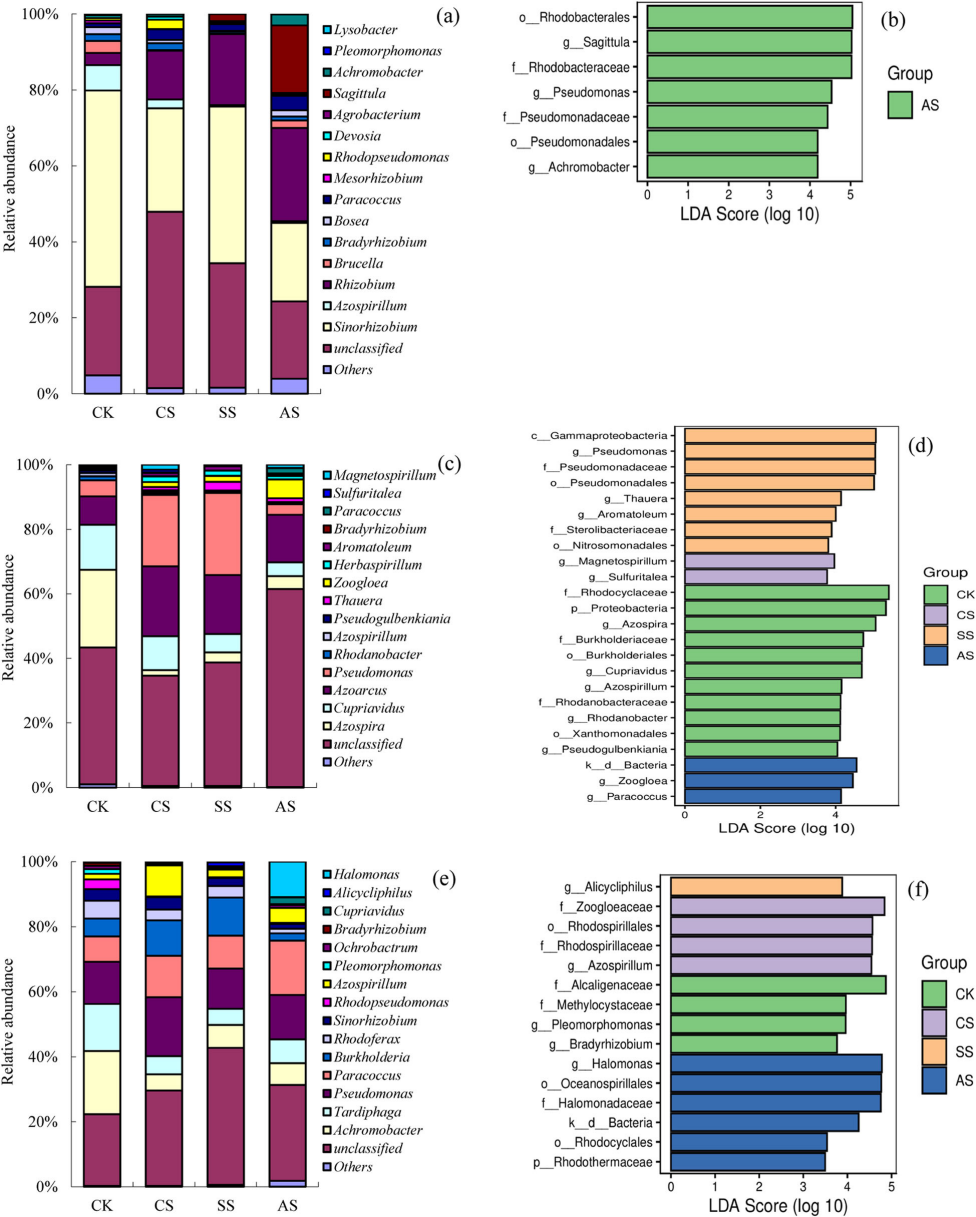
Genus-level composition (**a**, **c**, **e**) and linear discriminant analysis (LDA) effect size analysis (**b**, **d**, **f**) of bacterial communities based on denitrification functional genes under different types of salt and alkali stresses in a calcareous desert soil. Relative abundance (%) of genera in **a** *nirK*, **c** *nirS*, and **e** *nosZ* communities. LDA scores of dominant taxa in **b** *nirK*, **d** *nirS*, and **f** *nosZ* communities. CK, control treatment without salt or alkali stress; CS, NaCl stress treatment; SS, Na_2_SO_4_ stress treatment; AS, Na_2_CO_3_ + NaHCO_3_ stress treatment

The genotypes of denitrifying communities were significantly affected by saline and alkaline stresses (Fig. 7). Figure 7a shows the composition of the *nirK*-type denitrifier communities. The dominant genera included *Sinorhizobium* and *Rhizobium*, which together accounted for 40.10 to 59.81% of the relative abundance in all treatments. The highest relative abundance of *Sinorhizobium* was in CK, with the relative abundance 89.80% higher than that in CS, 25.57% higher than that in SS, and 148.92% higher than that in AS. However, the lowest relative abundance of *Rhizobium* was also observed in CK, with the relative abundance 75.22% lower than that in CS, 82.89% lower than that in SS, and 87.01% lower than that in AS. The other genera in the *nirK*-type denitrifier communities included *Azospirillum* (0.40 to 6.96%), *Brucella* (0.12 to 3.13%), *Bradyrhizobium* (0.56 to 1.83%), *Bosea* (0.19 to 1.81%), *Paracoccus* (1.03 to 3.81%), *Mesorhizobium* (0.04 to 0.74%), *Rhodopseudomonas* (0.37 to 2.52%), *Devosia* (0.01 to 0.77%), *Agrobacterium* (0.01 to 0.30%), *Sagittula* (0.04 to 17.73%), *Achromobacter* (0.01 to 2.89%), *Pleomorphomonas*(0.00 to 0.14%), and *Lysobacter*(0.00 to 0.01%). In addition, the relative abundance of *Azospirillum* and *Brucella* in CK was significantly higher than that in CS, SS, and AS. Dominant *nirK*-type denitrifier genera appeared only in AS (Fig. 7b), and the relative abundances of *Sagittula* (17.73%), *Achromobacter* (2.89%), and *Pseudomonas* (0.14%) were significantly higher than those in other treatments.

In the *nirS*-type communities, the dominant genera included *Azospira*, *Cupriavidus*, *Azoarcus*, and *Pseudomonas* (Fig. 7c). The four genera accounted for 26.34 to 56.09% of the total relative abundance in all treatments. The composition of *nirS*-type communities varied significantly among the saline and alkaline stress treatments. Compared with CK, the relative abundance of *Azospira* decreased significantly 24.02 to 1.71% and that of *Cupriavidus* 13.95 to 4.24% under saline and alkaline stresses. By contrast, the relative abundance of *Azoarcus* increased significantly 8.80 to 21.60% under saline and alkaline stresses. Compared with CK, the relative abundance of *Pseudomonas* increased significantly in CS and SS; however, there was no significant difference between AS and CK. The other genera in the *nirS*-type communities included *Rhodanobacter* (0.11 to 1.22%), *Azospirillum* (0.21 to 0.98%), *Pseudogulbenkiania* (0.18 to 0.78%), *Thauera* (0.57 to 2.71%), *Zoogloea* (0.51 to 5.85%), *Herbaspirillum* (0.47 to 1.68%), *Aromatoleum* (0.15 to 1.34%), *Paracoccus* (0.03 to 1.65%), *Sulfuritalea* (0.01 to 0.73%), *Bradyrhizobium* (0.08 to 0.23%), and *Magnetospirillum* (0.00 to 1.62%). Figure 7d shows the dominant *nirS*-type denitrifier taxa. The dominant genera in CK were *Azospira* (24.02%), *Cupriavidus* (13.95%), *Azospirillum* (0.98%), *Rhodanobacter* (1.22%), and *Pseudogulbenkiania* (0.78%). In CS, the dominant genera were *Sulfuritalea* (0.73%) and *Magnetospirillum* (1.62%). In SS, *Pseudomonas* (25.43%), *Thauera* (2.71%), and *Aromatoleum* (1.34%) were the dominant genera, and in AS, *Zoogloea* (5.85%) and *Paracoccus* (1.65%) were dominant.

In the *nosZ*-type communities, the dominant genera included *Achromobacter*, *Tardiphaga*, *Pseudomonas*, *Paracoccus*, and *Burkholderia* (Fig. 7e)*.* The five genera accounted for 46.31 to 60.18% of the total relative abundance in all treatments, with the relative abundance of all > 1%. The relative abundances of *Achromobacter* and *Tardiphaga* were higher in CK than in CS, SS, and AS. However, the relative abundance of *Paracoccus* was lower in CK than in CS, SS, and AS. In CS and SS, the relative abundance of *Burkholderia* was significantly higher than that in CK and AS, with the lowest relative abundance in AS. However, the relative abundance of *Pseudomonas* was not significantly different among the four treatments. The other genera in *nosZ*-type communities included *Rhodoferax* (1.40 to 5.51%), *Sinorhizobium* (1.54 to 3.83%), *Rhodopseudomonas* (0.19 to 2.93%), *Azospirillum* (1.72 to 9.50%), *Pleomorphomonas* (0.00 to 1.44%), *Ochrobactrum* (0.25 to 0.99%), *Bradyrhizobium* (0.24 to 0.96%), *Cupriavidus* (0.24 to 2.15%), *Halomonas* (0.00 to 11.08%), and *Alicycliphilus* (0.00 to 1.22%), which were in all treatments. By pairwise comparison among different treatments, *Pleomorphomonas* (1.44%) and *Bradyrhizobium* (0.96%) were dominant in CK, *Azospirillum* was dominant in CS (9.50%), *Alicycliphilus* was dominant in SS (1.22%), and *Halomonas* (11.08%) was dominant in AS (Fig. 7f).

There were some common genera among *nirK*-type, *nirS*-type, and *nosZ*-type denitrifier communities. The genera *Achromobacter* and *Alcaligenes* were in both *nirK*-type and *nosZ*-type denitrifier communities. The *nirK* and *nosZ* genes were found in the genera *Aromatoleum*, *Azoarcus*, *Cupriavidus*, and *Herbaspirillum*. There were six denitrifiers that had *nirK* and *nosZ* genes, including *Brucella*, *Mesorhizobium*, *Ochrobactrum*, *Pleomorphomonas*, *Rhodopseudomonas*, and *Sinorhizobium*. In addition, all three *nirK*, *nirS*, and *nosZ* genes were identified only in the genera *Azospirillum*, *Bradyrhizobium*, *Paracoccus*, *Pseudomonas*, and *Rhodanobacter*.

### Redundancy analysis

**Fig. 8:**
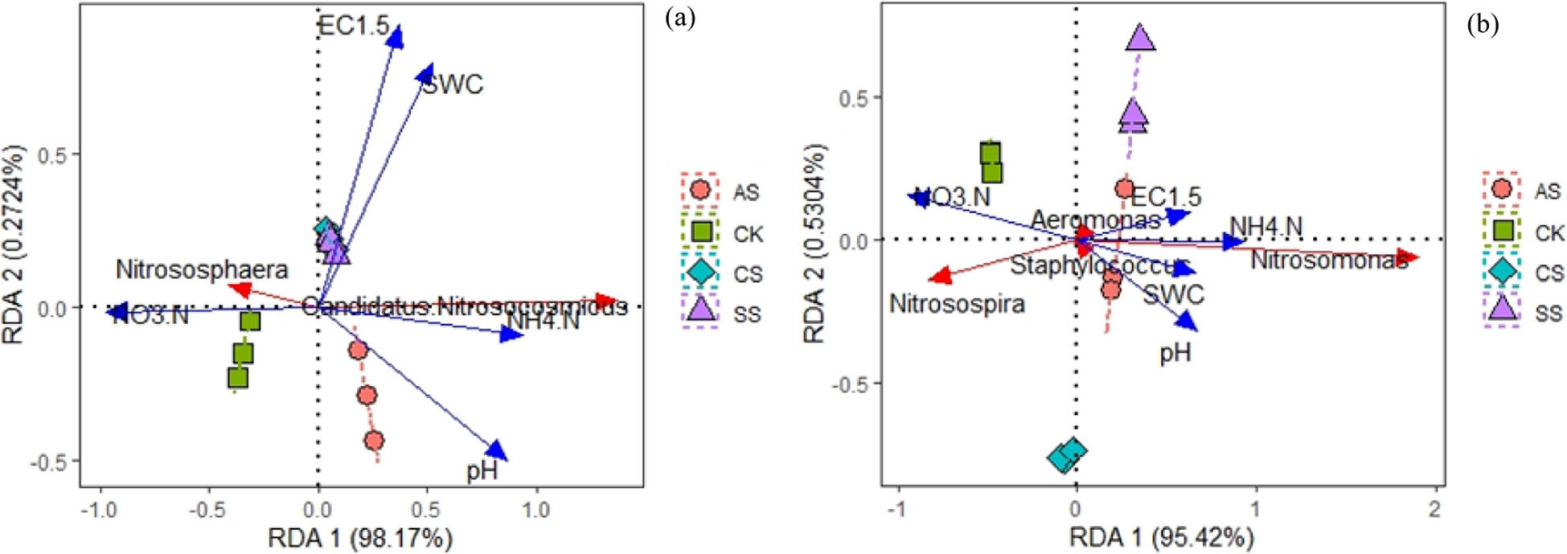
Redundancy analyses (RDA) of the correlations between the genera of **a** *amoA*-AOA and **b** *amoA*-AOB communities and soil variables in a calcareous desert soil. CK, control treatment without salt or alkali stress; CS, NaCl stress treatment; SS, Na_2_SO_4_ stress treatment; AS, Na_2_CO_3_ + NaHCO_3_ stress treatment

Figure 8 shows the correlations between species with *amoA*-AOA and *amoA*-AOB genes at the genus level and soil properties. Figure 8a shows the correlation of *amoA*-AOA community structure with soil properties. Axis 1 and 2 explained 98.44% of the total variation. The CK samples were clearly separated from those of CS, SS, and AS along axis 1 (98.17%). However, there was no significant difference between CS and SS. The *amoA*-AOA community structure was significantly correlated with soil pH (variation explained, 71.31%; *P =* 0.001), SWC (variation explained, 19.50%, *P =* 0.001), and soil salinity (variation explained, 5.40%, *P =* 0.003). *Nitrososphaera* was positively correlated with NO_3_-N and negatively correlated with pH and NH_4_-N, whereas the correlations with *Candidatus Nitrosocosmicus* were the opposite. For *amoA*-AOB community structure, axis 1 and axis 2 together explained 95.95% of the total variation (Fig. 8b). The CK samples were clearly separated from those of CS, SS, and AS along axis 1 (95.42%). The *amoA*-AOB community structure was significantly correlated with SWC (variation explained, 35.61%, *P* = 0.001) and pH (variation explained, 37.18%, *P* = 0.001) but not with the other soil properties. *Nitrosospira* was positively correlated with NO_3_-N and negatively correlated with pH and NH_4_-N; whereas the correlations with *Nitrosomonas* were the opposite.

**Fig. 9:**
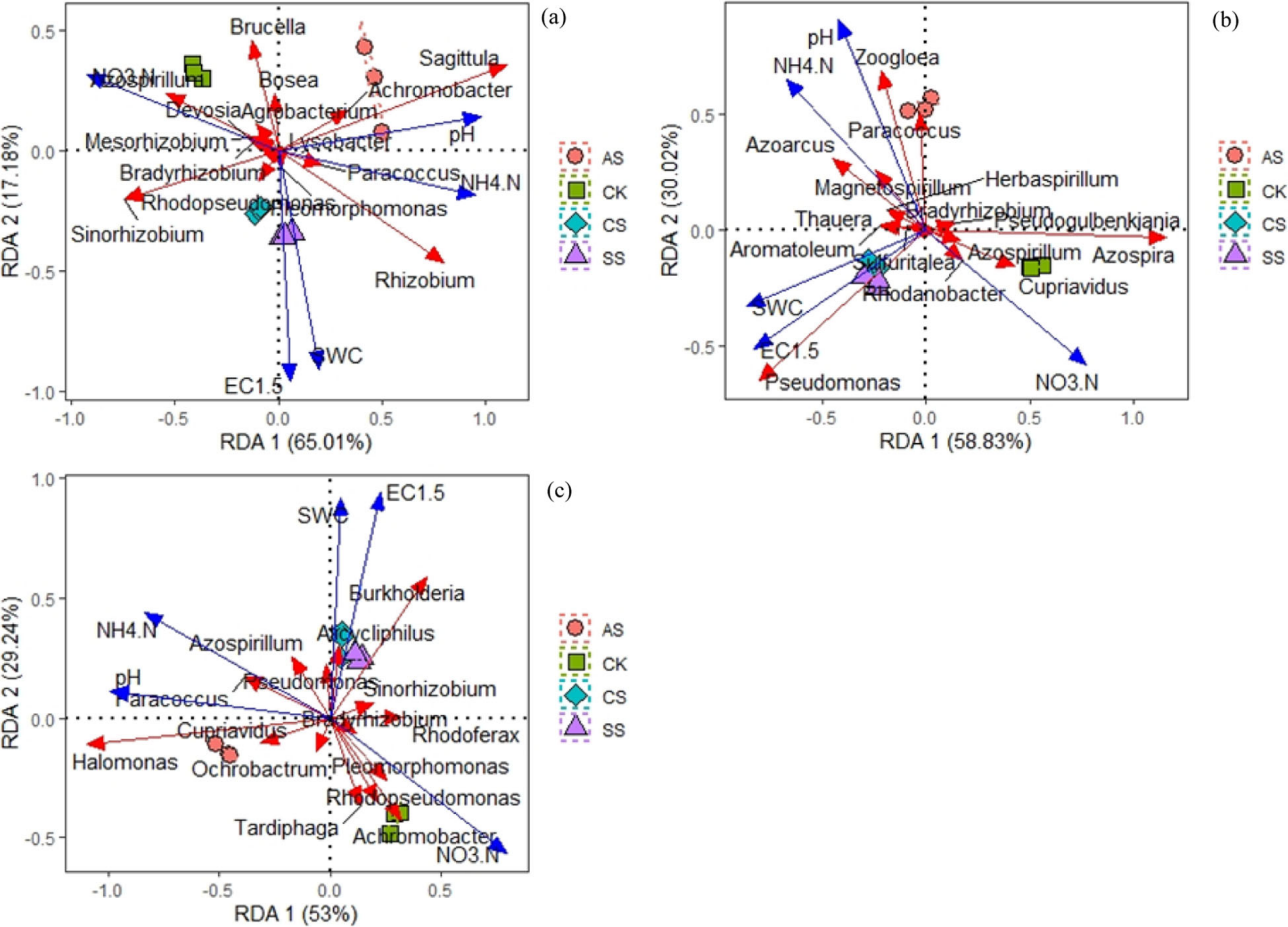
Redundancy analyses of the correlations between the genera of the denitrifier **a** *nirK*, **b** *nirS*, and **c** *nosZ* communities and soil variables in a calcareous desert soil. CK, control treatment without salt or alkali stress; CS, NaCl stress treatment; SS, Na_2_SO_4_ stress treatment; AS, Na_2_CO_3_ + NaHCO_3_ stress treatment

Figure 9 shows the correlations between species with *nirK*, *nirS*, and *nosZ* genes at the genus level and soil properties. Axis 1 and 2 explained 82.19% of the total variation in the composition of *nirK*-type denitrifier communities (Fig. 9a). The *nirK*-type denitrifier community structure was significantly correlated with pH (variation explained, 58.61%, *P* = 0.001), SWC (variation explained, 8.37%, *P* = 0.002), and salinity (variation explained, 7.90%, *P* = 0.03). *Sinorhizobium*, *Rhizobium*, *Sagittula*, and *Paracoccus* were negatively correlated with pH and NH_4_-N and positively correlated with NO_3_-N; whereas the correlations with *Azospirillum* and *Mesorhizobium* were the opposite. *Bradyrhizobium*, *Devosia*, and *Agrobacterium* were positively correlated with NO_3_-N and negatively correlated with NH_4_-N. *Brucella* and *Bosea* were negatively correlated with SWC and salinity. *Achromobacter* was positively correlated with NH_4_-N and pH. The other genera did not separate and were concentrated at the original point.

For *nirS*-type denitrifier community structure, axes 1 and 2 explained 88.85% of the total variation (Fig. 9b). The *nirS*-type denitrifier community structure was significantly correlated with SWC (variation explained, 41.99%, *P* = 0.001), salinity (variation explained, 42.90%, *P* = 0.009), and pH (variation explained, 28.49%, *P* = 0.001), but not with the other soil properties. *Cupriavidus*, *Azospira*, *Rhodanobacter*, and *Azospirillum* were negatively correlated with pH and NH_4_-N and positively correlated with NO_3_-N, whereas the correlations with *Zoogloea* and *Paracoccus* were the opposite. *Azoarcus*, *Pseudomonas*, *Thauera*, *Herbaspirillum*, and *Aromatoleum* were positively correlated with SWC and salinity. *Pseudogulbenkiania* was negatively correlated with SWC, NH_4_-N, and salinity and positively correlated with NO_3_-N.

Axis 1 and 2 contributed 82.24% of the total variation in the structure of the *nosZ*-type denitrifier communities (Fig. 9c). The *nosZ*-type denitrifier community structure was significantly correlated with soil pH (variation explained, 47.42%, *P* = 0.001), salinity (variation explained, 21.61%, *P* = 0.003), and SWC (variation explained, 17.28%, *P* = 0.002), but not with the other soil properties. *Paracoccus* and *Halomonas* were positively correlated with pH and NH_4_-N and negatively correlated with NO_3_-N, whereas the correlations with *Rhodoferax* and *Sinorhizobium* were the opposite. *Achromobacter*, *Rhodopseudomonas*, and *Pleomorphomonas* were negatively correlated with SWC, salinity, pH, and NH_4_-N and positively correlated with NO_3_-N. *Burkholderia* and *Alicycliphilus* were positively correlated with SWC and salinity; whereas the correlations with *Ochrobactrum* were the opposite. *Tardiphaga* was negatively correlated with SWC, salinity, and NH_4_-N and positively correlated with NO_3_-N. *Bradyrhizobium* was negatively correlated with SWC, pH, and NH_4_-N and positively correlated with NO_3_-N. *Cupriavidus* was positively correlated with NH_4_-N and NO_3_-N.

## Discussion

Soil salinization is a worldwide problem and a major challenge to sustaining soil quality. It is an important factors limiting agriculture production in arid regions. Soil salinity mainly causes damage to plants through ion toxicity and osmotic stress, and the inhibition of plant growth is the most common physiological response in a saline and alkaline habitat [35]. In this study, saline and alkaline stresses significantly inhibited cotton growth. The inhibition might have been due to the toxicity of Na ions with salt stress [36] and the increase in pH and disturbance of plant nutrition and metabolism with alkaline stress [37]. Salinity adversely affects soil physicochemical properties, which in turn, affect ecosystem nutrient cycling and especially the key transformations of N [38, 39]. In this study, NH_4_-N content increased significantly but NO_3_-N content decreased significantly under saline and alkaline stresses (Fig. 1d, e), which might be explained by the inhibition of soil nitrification due to the increase in soil salinity [40]. Saline and alkaline stresses also significantly inhibited soil PNR in this study (Fig. 1f). Akhtara et al. and He et al. also found that nitrification rates decrease with increases in soil salinity [11, 41]. Thus, these results suggest that saline and alkaline stresses inhibit the conversion of NH_4_-N to NO_3_-N, the key microbial process associated with nitrification.

Salinity stress affects soil biological properties by decreasing the abundance and diversity of microbial communities [42]. Moreover, soil salinization is usually accompanied by alkalization, which causes further serious deterioration of soil properties [43]. Microbially mediated soil N transformations, such as nitrification and denitrification, are also influenced by changes in salinity [44, 45]. Ammonia oxidation is the first and rate-limiting step in nitrification, with AOB and AOA the primary microbial groups involved [10]. Li et al. found that the copies of *amoA*-AOB and *amoA*-AOA are negatively correlated with soil salinity [19]. In this study, saline and alkaline stresses significantly decreased th*e* gene copy numbers of *amoA*-AOA and *amoA*-AOB (Fig. 2a, b). This result suggests that the increases in salinity and pH caused by saline and alkaline stresses are not suitable for the growth and reproduction of AOB and AOA. However, the gene copies of *amoA*-AOB were higher than those of *amoA*-AOA in this study. One explanation is that AOB prefer neutral pH and high-N agricultural soils, whereas AOA dominate in acidic or low nutrient-content soils [14]. Others have also observed higher abundance of AOB than that of AOA in saline and alkaline soils [46, 47] and AOA as the dominant microbial group in acidic soils [48]. Moreover, Nicol et al. reported that *amoA*-AOB copies decrease with a decrease in soil pH, whereas those of *amoA*-AOA decrease with an increase in pH (from 4.9 to 7.5) [9]. Li et al. reported a pH range of 5.0 to 7.0 for AOA enriched from activated sludge, with the optimum pH at 6.0 [49]. The results collectively suggest that soil pH has different effects on the ecological sites of AOB and AOA under different environmental conditions. In this study, the higher *amoA*-AOB copy numbers indicated that AOB was the dominant group in the ammonia-oxidizing community and the major contributor to ammonia oxidation in the saline and alkaline soils. In addition, the copy numbers *amoA*-AOB and *amoA*-AOA were significantly positively related to soil PNR (Fig. 3), suggesting that AOB and AOA participated in nitrification in the saline and alkaline soils. In addition, the *amoA*-AOA/*amoA*-AOB ratio was significantly lower under saline and alkaline stresses than that in the control soil. The decrease in the *amoA*-AOA/*amoA*-AOB ratio of the ammonia-oxidizing community indicated there was selective pressure against AOA under saline and alkaline stresses. Collectively, these results support the hypothesis that AOB are adapted to alkaline to neutral pH soils, whereas AOA are adapted to acidic soils. Nevertheless, the *amoA*-AOA/*amoA*-AOB ratio alone does not provide sufficient information to determine which of the two ammonia-oxidizing groups is functionally dominant in ammonia oxidation [50, 51]. The decreases in *amoA* gene copies might indicate lower potential soil nitrification under the saline and alkaline stresses. Indeed, the copy numbers of *amoA*-AOB and *amoA*-AOA *amoA* in this study were highly related to PNR (*P <* 0.001 and *P =* 0.005, respectively; Fig. 3). The positive linear relations indicated that *amoA*-AOB and *amoA*-AOA were most likely important in explaining the variation in PNR in this soil. However, because the number of *amoA*-AOB copies was higher than that of *amoA*-AOA, the AOB community might have played a more important role in soil nitrification. Moreover, in correlation analyses, *amoA*-AOB and *amoA*-AOA copies were positively correlated with NO_3_-N content (Table 1), further indicating that AOA and AOB contributed to nitrification.

The effects of salinity on the structure of AOB and AOA communities have been investigated in many previous studies [19, 20]. Salinity significantly alters the structure of AOA and AOB communities in wetland soil [52]. In this study, CS decreased the Shannon index of the *amoA*-AOB community, and AS decreased the Chao 1 index of the *amoA*-AOA and *amoA*-AOB communities (Table 3). Dang et al. also found that salinity decreases the diversity of the AOB community [53]. The NMDS analysis (Fig. 5) showed clear separation of communities under saline and alkaline stresses from those in control soil, suggesting that the changes in *amoA*-AOB and *amoA*-AOA communities might be partially attributed to the low concentrations of soil mineral N and relatively high pH values associated with saline and alkaline stresses. In addition, in the *amoA*-AOA communities in this study, the main genera were *Nitrososphaera* and *Candidatus Nitrosocaldus*. Saline and alkaline stresses significantly increased the relative abundance of *Candidatus Nitrosocaldus*, indicating it was strongly tolerant of saline and alkaline stresses. According to Lehtovirta-Morley et al. *Candidatus Nitrosocosmicus* (AOA), in a *Nitrososphaera* sister cluster, was first isolated from a near-neutral pH agricultural soil, suggesting its potential contribution to ammonia oxidation in neutral pH soils [54]. Wu et al. also reported that alkaline soil is suitable for the growth of *Candidatus Nitrosotalea* (AOA), which shows strong adaptability to pH variation. In the *amoA*-AOB communities, the dominant genera were *Nitrosospira* and *Nitrosomonas* [55]. Saline and alkaline stresses significantly increased the relative abundance of *Nitrosomonas* but significantly decreased that of *Nitrosospira.* By contrast, Sahan and Muyzer found that *Nitrosospira* is enriched in a high-salt environment, whereas *Nitrosomonas* is enriched in a low- or medium-salt environment [56]. According to the Lefse analysis, the dominant genera in CK were *Nitrososphaera* and *Nitrosospira*, whereas *Nitrosomonas* was significantly enriched in SS and *Candidatus Nitrosocosmicus* was significantly enriched in AS. In this study, the variations in the *amoA*-AOA community were closely associated with salinity, SWC, and pH, whereas the variations in the *amoA*-AOB community were only significantly correlated with SWC and pH. Hu et al. also found that the communities of AOA and AOB were positively correlated with pH [57]. Nevertheless, we could not accurately determine the contributions of the *amoA*-AOA and *amoA*-AOB communities to nitrification, which need to be investigated further.

Saline and alkaline stresses alter soil physicochemical properties, thereby affecting microbial processes. In this study, the copies of *nirK* decreased in CS; however, the copies increased significantly in SS and AS. Stress from NaCl can inhibit denitrification activity [58], and decrease the abundance of denitrifying bacteria [59]. Wang et al. also reported that salinity significantly decreases the abundance of *nirK* genes [28]. In this study, saline and alkaline stresses significantly increased the copies of *nirS* and *nosZ*. Franklin et al. found that the number of denitrifying bacteria also increases with salinity in a beach wetland [60]. One explanation is that saline and alkaline stresses increase soil water content and cause poor soil aeration. Because the *nosZ* gene is sensitive to oxygen [61], its activity can be inhibited under aerobic conditions [62]. Therefore, with reduced soil aeration after irrigation with saline water, the growth of bacteria with the *nosZ* genotype may be stimulated [63]. Emissions of N_2_O are inversely related to *nosZ* gene expression [12]. In addition, an increase in *nosZ* gene copies indicates that the denitrification process is more complete, leading to N_2_ as the end product [64]. Moreover, the number of copies of *nirK* and *nosZ* was significantly lower than that of *nirS*. Similarly, Mosier and Francis and Santoro et al. also found that copies of *nirS* are higher than those of *nirK* [65, 66]. Francis et al. found that the *nirS* gene can increase in richness in low or medium salinity regions, significantly changing community structure and also indicating that *nirS* is more important in denitrification than *nirK* or *nosZ* [60]. In our study, the copies of *nirS* were positively correlated with pH and NH_4_-N (Table 1). Morales et al. also found that *nirS* copies are significantly positively correlated with NH_4_-N content [67]. The copies of *nosZ* were positively correlated with SWC and EC_1:5_ (Table 1). The copies of both *nirS* and *nosZ* were negatively correlated with NO_3_-N; whereas the copies of *nirK* were not significantly correlated with soil properties. These results indicate that *nirS*- and *nosZ*-type denitrifiers are more sensitive than *nirK*-type denitrifiers to saline and alkaline stresses.

Changes in the copies of denitrifying bacteria under saline and alkaline stresses likely alter community diversity. In this study, CS and SS decreased the Chao1 index of *nirK*, but saline and alkaline stresses significantly increased that of *nirS* and *nosZ* (Table 3). These results suggested that neutral salt (CS and SS) stress reduced the abundance of *nirK* but AS increased that of *nirS* and *nosZ*. In addition, saline and alkaline stresses decreased the Shannon index of *nirK*, whereas CS and SS increased the Shannon index of *nirS*. These results suggested that saline and alkaline stresses reduced the diversity of *nirK* but neutral salt (CS and SS) stress increased that of *nirS*. Thus, saline and alkaline stresses altered the community structure of denitrifying bacteria. The *nosZ* gene is considered to be relatively stable [68], and in this study, saline and alkaline stresses had no significant effect on its Shannon index. However, this result is in contrast to that of Yang et al. who reported that salinity is positively correlated with the diversity of *nosZ* genes [69]. The NMDS analysis also showed that saline and alkaline stresses significantly altered the community structure of denitrifying bacteria.

In this study, compared with CK, the saline and alkaline stresses altered the community structure of *nirK*-type denitrifiers. The dominant *nirK*-type denitrifiers were *Sinorhizobium* and *Rhizobium*, similar to the observations by Tang et al. [70]. Saline and alkaline stresses significantly increased the relative abundance of *Rhizobium*, whereas that of *Sinorhizobium* significantly decreased, indicating that *Rhizobium* was strongly tolerant of saline and alkaline stresses. In the *nirS*-type communities, the dominant genera were *Azospira*, *Cupriavidus*, *Azoarcus*, and *Pseudomonas*. Saline and alkaline stresses significantly decreased the abundance of *Azospira* and *Cupriavidus*, whereas that of *Azoarcus* significantly increased*.* In addition, neutral salt (CS and SS) stress significantly increased the relative abundance of *Pseudomonas*. These results indicated that *Azoarcus* was strongly tolerant of saline and alkaline stresses and that *Pseudomonas* was strongly tolerant of neutral salt stress. In the *nosZ*-type communities, the dominant genera were *Achromobacter*, *Tardiphaga*, *Pseudomonas*, *Paracoccus*, *and Burkholderia.* Saline and alkaline stresses significantly decreased the abundance of *Achromobacter* and *Tardiphaga*, whereas that of *Paracoccus* significantly increased*.* In addition, neutral salt (CS and SS) stress significantly increased the relative abundance of *Burkholderia*, whereas alkaline stress significantly decreased it. These results indicated that *Paracoccus* was strongly tolerant of saline and alkaline stresses and that *Burkholderia* was strongly tolerant of neutral salt stress. *Burkholderia* also imparts some degree of tolerance in plants to other abiotic stresses such as drought, metal toxicity, and high temperature [71]. The AS treatment had the most potential biomarker species of *nirK* and *nosZ* genes, whereas the CS and AS treatments had the fewest potential biomarker species of the *nirS* gene. The fewest potential biomarkers species of the *nosZ* gene were in the SS treatment. These results indicated that saline and alkaline stresses affected the structure of different denitrifying bacteria communities to varying degrees. According to RDA, the variations in denitrifier communities were largely explained by salinity, SWC, and pH. Denitrifier community structure is also significantly correlated with salinity, pH, and SWC in previous studies [27, 72].

Farmland soil is the most important source of N_2_O emissions to the atmosphere, and the microbial processes involved in the N cycle are the primary drivers of those emissions [73]. Henry et al. and Zhao et al. reported that *nirS* and *nirK* genes are responsible for the microbial production of N_2_O; whereas the *nosZ* gene is responsible for reducing N_2_O to N_2_ [74, 75]. Thus, the denitrifier communities are critical in regulating N_2_O emissions. Moreover, nitrification may be the main source of N_2_O in arid regions [76] Therefore, soil N_2_O emissions include potential contributions from ammonia-oxidizing bacteria and archaea, which release N_2_O during the nitrification–denitrification process or through links to that process. Thus, the relative contributions of nitrification and denitrification to N_2_O production in saline and alkaline soils should be considered in further research.

## Conclusion

The results of pot experiment support our hypothesis that saline and alkaline stresses changed the abundance and composition of nitrifier and denitrifier community. Saline and alkaline stresses decreased the copy numbers of amoA-AOA and amoA-AOB but increased those of nirS and nosZ, and there were more gene copies of *amoA*-AOB than of *amoA*-AOA and more gene copies of *nirS* than of *nirK* and *nosZ*. The PNR was positively linearly related to the copy numbers of both *amoA-*AOB and *amoA*-AOA. In addition, saline and alkaline stresses greatly affected the richness, diversity, and structure of nitrifier and denitrifier communities. Saline and alkaline stresses led to increases in the relative abundance of *Candidatus Nitrosocosmicus*, *Nitrosomonas*, *Rhizobium*, *Azoarcus*, and *Paracoccus* but decreases in the relative abundance of *Nitrososphaera*, *Nitrosospira*, *Sinorhizobium*, *Azospira*, *Cupriavidus*, *Achromobacter*, *Tardiphaga*, and *Rhodoferax*. The pH and SWC were main drivers of changes in the abundance in *amoA*-AOA and denitrifier communities, whereas *amoA*-AOB community structure was only significantly correlated with SWC and pH. Therefore, *amoA*-AOA and *amoA*-AOB communities contribute to nitrification in alluvial gray desert soil and that the *nirS* community may have a more important role in denitrification than *nirK* and *nosZ* communities. The present study proposed that a theoretical basis for the efficient use of N fertilizers and rational N management in saline or alkaline soils in arid areas.

## Methods

### Experimental site and soil description

Surface soils (0 to 30 cm) were collected from a cotton field (*Gossypium hirsutum* L.) at the experimental station of Shihezi University in Shihezi, Xinjiang Province, China (44°18′N, 86°02′E). The climate is temperate arid zone with a mean annual temperature of 7.8 °C, precipitation of 210 mm, and evaporation of 1660 mm, with little annual variation. The soil was collected from multiple points in an unfertilized cotton field in March 2019. The soil is classified as calcareous desert soil (Calcaric Fluvisol in the FAO/UNESCO system) with a loam texture. The soil physicochemical properties were the following: electric conductivity (EC_1:5_), 0.35 dS·m^− 1^; pH, 7.86; organic matter, 14.9 g·kg^− 1^; alkaline N, 41.2 mg·kg^− 1^; available P, 10.6 mg·kg^− 1^; and available K, 248 mg·kg^− 1^.

### Experimental design

A pot experiment was performed in the experiment station greenhouse at Shihezi University. The cotton was planted on 25 April 2020, and seedlings were selected at the two-leaf stage, with four uniform seedlings kept in each pot. The pot experiment was conducted from 25 April 2020 to 10 September 2020. During the experiment, the maximum temperature and minimum temperature of the greenhouse were 15.8 °C and 41.6 °C, respectively.

According to the salt components and pH in most of the salt-affected soils in Xinjiang, China, three common types of salt-affected soils were obtained by adding chloride as NaCl (chloride stress, CS), sulfate as Na_2_SO_4_ (sulfate stress, SS), or carbonate as Na_2_CO_3_ + NaHCO_3_ (alkaline stress, AS) to the sampled soil. The control (CK) soil had no saline or alkaline stress. The soil EC_1:5_ and pH values of the different saline and alkaline stress treatments and their salinization or alkalization degree are shown in Table 5.

**Table 5:** Soil EC_1:5_, pH values, and Na^+^ concentration in different saline and alkaline stress treatments in a calcareous desert soil

Treatment	Salinity and alkalinity	EC_1:5_ (dS·m^−1^)	pH (1:2.5)	Na^+^ concentration (g/kg)
Control (CK)	No additional salinization or alkalization	0.35	8.16	0.060
NaCl (CS)	Moderate salinization	1.39	8.43	0.886
Na_2_SO_4_ (SS)	Moderate salinization	2.01	8.19	0.827
Na_2_CO_3_ + NaHCO_3_ (AS)	Moderate alkalization	0.63	9.92	0.466

The field-collected soil was naturally dried and then crushed and sieved (2-mm pore size). Solutions of NaCl, Na_2_SO_4_, or Na_2_CO_3_ + NaHCO_3_ (weight ratio 1:1) were added to the soil to produce a supersaturated state (the same volume of deionized water was added to the control soil). The NaCl, Na_2_SO_4_, Na_2_CO_3_ + NaHCO_3_ addition amount were 4.0 g/kg, 6.0 g/kg, and 1.5 g/kg, respectively. After mixing evenly, the treated soil was left to stand for 1 month to ensure homogeneous distribution of salt. Then, the three treatment soils were naturally dried, crushed, and passed through a 2-mm sieve.

Non-draining soil pots with 35-cm internal diameter and 60-cm height were used. The treated soil was added to a bulk density of 1.25 g·cm^− 3^, with 60.0 kg per soil pots. The experiment was a completely randomized block design with three replications per treatment. The pots were drip-irrigated, and the emitters (and columns) were 0.4 m apart with a discharge rate (pressure compensated) of 2.1 L·h^− 1^. The drip irrigation pipe was laid flat on the surface of the soil pots, with each soil pot supplied by one emitter fixed at the center at the top of the pot. During the cotton growing season, the pots were irrigated 12 times. The irrigation interval was seven to 10 days, and 52 L of irrigation water was added per pot. The pots were all irrigated on the same dates. A flow meter was used to measure the amount of water applied.

The same amount of N (1,350 kg ha^− 1^, 13.73 g per pot), P_2_O_5_ (105 kg ha^− 1^, 1.07 g per pot), and K_2_O (60 kg ha^− 1^, 0.61 g per pot) was applied in all treatments. The N fertilizer was applied through the drip irrigation system during the cotton growing season. Consistent with local practices, urea was the N source. The N fertilizer was applied in six equal amounts 53, 64, 72, 81, 90, and 99 days after planting. All pots were fertilized with P_2_O_5_ and K_2_O before sowing.

### Cotton sampling

To determine the dry matter of cotton, three representative cotton plants were selected in each treatment on 5 August 2020 (103 days after planting). The roots, stems, and leaves was washed with distilled water and then dried in an oven at 70 °C for 48 h, weighed.

### Soil sampling

Soil samples were collected from the 0 to 20 cm layer from three pots per treatment on 5 August 2020 (103 days after planting). The samples were stored with ice packs and transported to the laboratory. Soils were passed through a 2-mm sieve, after which each soil sample was divided into three subsamples. One subsample was immediately flash-frozen in liquid nitrogen and stored at − 80 °C for total DNA extraction. One subsample was stored immediately at 4 °C to determine soil water content (SWC), soil mineral N content, and potential nitrification rate (PNR). The remaining subsample was air-dried to determine soil salinity and pH.

### Soil analyses

Soil water content was determined gravimetrically by oven drying at 105 °C until constant weight. Soil NH_4_-N and NO_3_-N were extracted with 2 mol L^− 1^ KCl (5 g of soil in 50 mL of KCl solution) on a horizontal shaker for 1 h at 220 rpm and then measured by a Smart Chem140 auto discrete Analyzer (Westco Scientific, Danbury, Connecticut, USA). Soil salinity and pH were determined with an MP521 lab pH/conductivity meter in a soil: water ratio of 1:5 and 1:2.5, respectively. As an index of the size of active nitrifier populations, soil potential nitrification rate (PNR) was determined using the method described by Kurola et al. [77]. In brief, 5 g of fresh soil was put into 50 mL centrifuge tubes containing 20 mL of phosphate buffer saline solution with 1 mmol L^− 1^ (NH_4_)_2_SO_4_. To inhibit nitrite oxidation, potassium chlorate (KClO_3_) was added to the tubes at a final concentration of 10 mmol L^− 1^. After incubation for 24 h in the dark at the room temperature of 25 °C, nitrite (NO_2_-N) was extracted with 5 mL of 2 M KCl and determined spectrophotometrically at 545 nm with N-(1-naphthyl) ethylenediamine dihydrochloride.

### DNA extraction, qPCR assay, and pyrosequencing

Soil microbial DNA was extracted using a Power Soil™ DNA Isolation Kit (Mo Bio Laboratories Inc., USA) following the manufacturer’s instructions and then stored at − 80 °C. The DNA concentration and purity were measured using a UV-vis spectrophotometer (Thermo Fisher Scientific, Waltham, MA, USA) and agarose gel electrophoresis, respectively. After which, the DNA was stored at − 20 °C.

The abundances of *amoA*-AOA, *amoA*-AOB, *nirK*, *nirS*, and *nosZ* were determined by real-time qPCR. Shanghai Personal Biotechnology Co., Ltd. (Shanghai, China) performed the qPCR on a CFX96 Optical Real-Time Detection System (Bio-Rad Laboratories, USA). Target plasmids were constructed with PMD-18 plasmids (TaKaRa, Tokyo, Japan), and the correct gene inserts were chosen. The qPCR reaction was performed in triplicate in a 20-μL reaction system containing 10 μL of 2× SYBR Green qPCR Master Mix (Applied Biosystems, Foster City, CA, USA), 2 μL of DNA template, 1 μL of each primer, and 6 μL of ddH_2_O. After qPCR, the gene copy numbers of nitrification and denitrification genes were normalized by the amount of soil based on the dilution rates and the volumes of the DNA used in the qPCR. Table 6 lists detailed conditions for PCR amplification. The numbers of copies of the target genes were calculated from standard curves.

**Table 6:** Primers and thermal profiles used for real-time quantitative PCR of the different nitrifying and denitrifying genes in bacterial communities in a calcareous desert soil

Target gene	Primer	Sequence (5′–3′)	Thermal profile	References
*amoA*-AOA	Arch-amoAF	5′-STAATGGTCTGGCTTAGACG-3’	95 °C for 5 min; 40 cycles of 95 °C for 10 s, 55 °C for 20 s, and 72 °C for 30 s.	Hu et al. [78]
	Arch-amoAR	5′-GCGGCCATCCATCTGTATGT-3’		
*amoA*-AOB	amoA-1F	5′-GGGGTTTCTACTGGTGGT-3’	95 °C for 5 min; 40 cycles of 95 °C for 10 s, 55 °C for 20 s, and 72 °C for 30 s.	Ebie et al. [79]
	amoA-2R	5′- CCCCTCKGSAAAGCCTTCTTC − 3’		
*nirK*	F1aCu	5′-ATCATGGTSCTGCCGCG-3′	95 °C 2 min, 1 cycle; 95 °C 20 s, 63 °C 30 s, 72 °C 30 s, 85 °C 10 s, 35 cycles.	Hallin and Lindgren [80]
	R3Cu	5′-GCCTCGATCAGRTTGTGGTT-3′		
*nirS*	cd3aF	5′-GTSAACGTSAAGGARACSGG-3’	95 °C 2 min, 1 cycle; 95 °C 45 s, 55 °C 45 s, 72 °C 45 s, 85 °C 20 s, 40 cycles.	Dong et al. [81]
	R3cd	5′-GASTTCGGRTGSGTCTTGA-3’		
*nosZ*	nosZ-1126F	5′-GGGCTBGGGCCRTTGCA-3’	95 °C 2 min, 1 cycle; 95 °C 20 s, 60 °C 30 s, 72 °C 30 s, 40 cycles.	Wu et al. [82]
	nosZ-1381R	5′-GAAGCGRTCCTTSGARAACTTG-3’		

High-throughput sequencing was used to analyze the composition and diversity of *amoA*-AOA, *amoA*-AOB, *nirK*, *nirS*, and *nosZ* gene-based bacterial communities. The primers were the same as those used in the qPCR. The 25-μL reaction system included 2 μL of DNA template, 1 μL of forward and reverse primer (10 μM), 5 μL of 5× Q5 reaction buffer, 5 μL of 5× Q5 High-Fidelity GC buffer, 0.25 μL of Q5 High-Fidelity DNA Polymerase (5 U μL^− 1^), 2 μL of (2.5 mM) dNTPs, and 8.75 μL of ddH_2_O. The thermal cycle reaction system for the genes used the following program: initial denaturation at 98 °C for 5 min; 35 cycles consisting of denaturation at 98 °C for 30 s, annealing at 55 °C for 30 s, and elongation at 72 °C for 45 s; and a final extension at 72 °C for 5 min. The PCR primers were purified with Agencourt AMPure Beads (Beckman Coulter, Indianapolis, IN, USA) and quantified using a PicoGreen dsDNA Assay Kit (Invitrogen, Carlsbad, CA, USA) according to the manufacturer’s instructions. After the individual quantification step, equivalent amounts of samples were mixed before high-throughput sequencing was performed using the Illumina MiSeq platform with MiSeq Reagent Kit v3 at Shanghai Aqu Biotechnology Co., Ltd. (Shanghai, China).

### Data analyses

All data are expressed as the mean ± standard deviation. One-way ANOVA was conducted using SPSS (IBM Software, Chicago, IL, USA). Tukey’s test was used to identify significant differences among means (*P* < 0.05). Pearson’s correlation analysis was used to test the correlations between PNR, abundance of genes, and soil properties. The sequence data were analyzed using QIIME (version 1.8.0) and R packages (v 3.5.0). The diversity and richness indices were calculated using an operational taxonomic unit (OTU) table in QIIME, the sequences were grouped into OTUs using a definition of 95% similarity. The visualization analysis of classification and abundance results was performed in MEGAN. Non-metric Multidimensional scaling (NMDS) was also conducted based on genus-level compositional profiles. Constrained ordination by redundancy analysis (RDA) in R (vegan, v 3.5.0) was used to elucidate relations between the structure of *amoA*-AOA, *amoA*-AOB, *nirK*, *nirS*, and *nosZ* gene-based communities and the soil physicochemical properties measured for each sample. Linear discriminant analysis effect size (LEfSe) was calculated in Visual Genomics to search for statistically different biomarkers between treatments.

## Data Availability

All sequences recovered by high-throughput sequencing have been deposited into NCBI Sequence Read Archive (SRA, https://www.ncbi.nlm.nih.gov/sra). The accession number is PRJNA727758 (http://www.ncbi.nlm.nih.gov/bioproject/727758), which includes 60 accession items (SAMN 19032946 – SAMN19033005).

## Abbreviations

SWC: Soil water content
EC: Electrical conductivity
PNR: Potential nitrification rate
One-way ANOVA: One-way analysis of variance
OTU: Operational taxonomic unit
RDA: Redundancy analysis
NMDS: Nonmetric multidimensional scaling
LEfSe: Linear discriminant analysis effect size

## References

[1] Li N, Zheng H, Cui J, Wang J, Liu H, Sun J, Liu T, Zhao H, Lai Y, Zou D (2019). Genome-wide association study and candidate gene analysis of alkalinity tolerance in japonica rice germplasm at the seedling stage. Rice..

[2] Wicke B, Smeets E, Dornburg V, Vashev B, Gaiser T, Turkenburg W, Faaij A (2011). The global technical and economic potential of bioenergy from salt-affected soils. Energy Environ Sci.

[3] Morton MJ, Awlia M, Al-Tamimi N, Saade S, Pailles Y, Negrão S, Tester M (2019). Salt stress under the scalpel–dissecting the genetics of salt tolerance. Plant J.

[4] Wu YP, Li YF, Zheng CY, Zhang YF, Sun ZJ (2013). Organic amendment application influence soil organism abundance in saline alkali soil. Eur J Soil Biol.

[5] Shi DC, Yin LJ (1993). Difference between salt (NaCl) and alkaline (Na_2_CO_3_) stresses on *Puccinellia tenuiflora* (*Griseb*.) Scribn et Merr. plants. Acta Bot Sin.

[6] Tejada M, Garcia C, Gonzalez JL, Hernandez MT (2006). Use of organic amendment as a strategy for saline soil remediation: influence on the physical, chemical and biological properties of soil. Soil Biol Biochem.

[7] Li C, Lei J, Zhao Y, Xu X, Li S (2015). Effect of saline water irrigation on soil development and plant growth in the Taklimakan Desert highway shelterbelt. Soil Tillage Res.

[8] Henderson SL, Dandie CE, Patten CL, Zebarth BJ, Burton DL, Trevors JT, Goyer C (2010). Changes in denitrifier abundance, denitrification gene mRNA levels, nitrous oxide emissions, and denitrification in anoxic soilmicrocosms amended with glucose and plant residues. Appl Environ Microbiol.

[9] Nicol GW, Leininger S, Schleper C, Prosser JI (2008). The influence of soil pH on the diversity, abundance and transcriptional activity of ammonia oxidizing archaea and bacteria. Environ Microbiol.

[10] Gleeson DB, Müller C, Banerjee S, Ma W, Siciliano SD, Murphy DV (2010). Response of ammonia oxidizing archaea and bacteria to changing water filled pore space. Soil Biol Biochem.

[11] Akhtar M, Hussain F, Ashraf MY, Qureshi TM, Akhter J, Awan AR (2012). Influence of salinity on nitrogen transformations in soil. Commun Soil Sci Plant Anal.

[12] Harter J, Krause HM, Schuettler S, Ruser R, Fromme M, Scholten T, Kappler A, Behrens S (2014). Linking N_2_O emissions from biochar–amended soil to the structure and function of the N–cycling microbial community. ISME J.

[13] Cui PY, Fan FL, Yin C, Song AL, Huang PR, Tang YJ, Zhu P, Peng C, Li TQ, Wakelin SA, Liang YC (2016). Long-term organic and inorganic fertilization alters temperature sensitivity of N_2_O emissions and associatedmicrobes. Soil Biol Biochem.

[14] Di HJ, Cameron KC, Sherlock RR, Shen JP, He JZ, Winefield CS (2010). Nitrous oxide emissions from grazed grassland as affected by a nitrification inhibitor, dicyandiamide, and relationships with ammonia-oxidizing bacteria and archaea. J Soil Sediments.

[15] He JZ, Shen JP, Zhang LM, ZhuY G, Zheng YM, Xu MG, Di H (2007). Quantitative analyses of the abundance and composition of ammonia-oxidizing bacteria and ammonia-oxidizing archaea of a Chinese upland red soil under long-term fertilization practices. Environ Microbiol.

[16] Lehtovirta-Morley LE, Stoecker K, Vilcinskas A, Prosser JI, Nicol GW (2011). Cultivation of an obligate acidophilic ammonia oxidizer from a nitrifying acid soil. Proc Natl Acad Sci.

[17] Jiang X, Hou X, Zhou X, Xin X, Wright A, Jia Z (2015). pH regulates key players of nitrification in paddy soils. Soil Biol Biochem.

[18] Shi Y, Liu X, Zhang Q (2019). Effects of combined biochar and organic fertilizer on nitrous oxide fluxes and the related nitrifier and denitrifier communities in a saline-alkali soil. Sci Total Environ.

[19] Li XR, Xiao YP, Ren WW, Liu ZF, Shi JH, Quan ZX (2012). Abundance and composition of ammonia-oxidizing bacteria and archaea in different types of soil in the Yangtze River estuary. J Zhejiang Univ Sci B.

[20] Guo H, Ma L, Liang Y, Hou Z, Min W (2020). Response of ammonia-oxidizing Bacteria and Archaea to long-term saline water irrigation in alluvial grey desert soils. Sci Rep.

[21] Wang YF, Gu JD (2014). Effects of allylthiourea, salinity, and pH on ammonia/ammonium-oxidizing prokaryotes in mangrove sediment incubated in laboratory microcosms. Appl Microbiol Biotechnol.

[22] Mosier AC, Francis CA (2008). Relative abundance and diversity of ammonia-oxidizing archaea and bacteria in the San Francisco Bay estuary. Environ Microbiol.

[23] Azziz G, Monza J, Etchebehere C, Irisarri P (2017). *nirS*- and *nirK*-type denitrifier communities are differentially affected by soil type, rice cultivar and water management. Eur J Soil Biol.

[24] Shah SA, Shah Z (2011). Changes in soil microbial characteristics with elevated salinity. Sarhad J Agric.

[25] Li J, Ye W, Wei D, Huu Hao N, Guo W, Qiao Y, Xu W, Du B, Wei Q (2018). System performance and microbial community succession in a partial nitrification biofilm reactor in response to salinity stress. Bioresour Technol.

[26] Deng YL, Ruan YJ, Zhu SM, Guo XS, Han ZY, Ye ZY, Liu G, Shi MM (2017). The impact of DO and salinity on microbial community in poly (butylene succinate) denitrification reactors for recirculating aquaculture system wastewater treatment. AMB Express.

[27] Ma L, Guo H, Min W (2019). Nitrous oxide emission and denitrifier bacteria communities in calcareous soil as affected by drip irrigation with saline water. Appl Soil Ecol.

[28] Wang H, Gilbert JA, Zhu Y, Yang X (2018). Salinity is a key factor driving the nitrogen cycling in the mangrove sediment. Sci Total Environ.

[29] Franklin RB, Morrissey EM, Morina JC (2017). Changes in abundance and community structure of nitrate-reducing bacteria along a salinity gradient in tidal wetlands. Pedobiologia..

[30] Li X, Gao D, Hou L, Liu M (2019). Salinity stress changed the biogeochemical controls on CH_4_ and N_2_O emissions of estuarine and intertidal sediments. Sci Total Environ.

[31] Ai C, Liang G, Sun J, He P, Tang S, Yang S, Zhou W, Wang X (2015). The alleviation of acid soil stress in rice by inorganic or organic ameliorants is associated with changes in soil enzyme activity and microbial community composition. Biol Fertil Soils.

[32] Bai J, Gao H, Xiao R, Wang J, Huang C (2012). A review of soil nitrogen mineralization as affected by water and salt in coastal wetlands: issues and methods. Clean–Soil, Air, Water.

[33] He F, Chen Q, Jiang R, Chen X, Zhang F (2007). Yield and nitrogen balance of greenhouse tomato(*Lycopersicum esculentum Mill.*) with conventional and site-specific nitrogen management in northern China. Nutr Cycl Agroecosyst.

[34] Mapanda F, Wuta M, Nyamangara J, Rees RM (2012). Nitrogen leaching and indirect nitrous oxide emissions from fertilized croplands in Zimbabwe. Nutr Cycl Agroecosyst.

[35] Wang N, Qiao W, Liu X, Shi J, Xu Q, Zhou H, Yan GT, Huang Q (2017). Relative contribution of Na^+^/K^+^ homeostasis, photochemical efficiency and antioxidant defense system to differential salt tolerance in cotton (Gossypium hirsutum L.) cultivars. Plant Physiol Biochem.

[36] Lokhande VH, Nikam TD, Patade VY, Ahire ML, Suprasanna P (2011). Effects of optimal and supra-optimal salinity stress on antioxidative defence, osmolytes and in vitro growth responses in *Sesuvium portulacastrum* L. Plant Cell, Tissue Organ Culture (PCTOC).

[37] Yang C, Chong J, Li C, Kim C, Shi D, Wang D (2007). Osmotic adjustment and ion balance traits of an alkali resistant halophyte Kochia sieversiana during adaptation to salt and alkali conditions. Plant Soil.

[38] Amini S, Ghadiri H, Chen CR, Marschner P (2016). Salt-affected soils, reclamation, carbon dynamics, and biochar: a review. J Soils Sediments.

[39] Ma T, Zeng W, Li Q, Wu J, Huang J (2016). Effects of water, salt and nitrogen stress on sunflower (*Helianthus annuus L*.) at different growth stages. J Soil Sci Plant Nutr.

[40] Bernhard AE, Bollmann A (2010). Estuarine nitrifiers: new players, patterns and processes. Estuar Coast Shelf Sci.

[41] He H, Zhen Y, Mi T, Fu L, Yu Z (2018). Ammonia-oxidizing Archaea and Bacteria differentially contribute to ammonia oxidation in sediments from adjacent waters of Rushan Bay, China. Front Microbiol.

[42] Canfora L, Bacci G, Pinzari F, Lo Papa G, Dazzi C, Benedetti A (2014). Salinity and bacterial diversity: to what extent does the concentration of salt affect the bacterial community in a saline soil?. PLoS One.

[43] Rengasamy P (2010). Soil processes affecting crop production in salt-affected soils. Funct Plant Biol.

[44] Hou L, Zheng Y, Liu M, Gong J, Zhang X, Yin G, You L (2013). Anaerobic ammonium oxidation (anammox) bacterial diversity, abundance, and activity in marsh sediments of the Yangtze estuary. J Geophys Res: Biogeosci..

[45] Deng FY, Hou LJ, Liu M, Zheng YL, Yin GY, Li XF, Lin XB, Chen F, Gao J, Jiang XF (2015). Dissimilatory nitrate reduction processes and associated contribution to nitrogen removal in sediments of the Yangtze estuary. J Geophys Res : Biogeosci..

[46] Magalhães CM, Machado A, Bordalo AA (2009). Temporal variability in the abundance of ammonia-oxidizing bacteria vs. archaea in sandy sediments of the Douro River estuary, Portugal. Aquat Microb Ecol.

[47] Keshri J, Mishra A, Jha B (2013). Microbial population index and community structure in saline–alkaline soil using gene targeted metagenomics. Microbiol Res.

[48] Zhang LM, Hu HW, Shen JP, He JZ (2012). Ammonia-oxidizing archaea have more important role than ammonia-oxidizing bacteria in ammonia oxidation of strongly acidic soils. ISME J.

[49] Li Y, Ding K, Wen X, Zhang B, Shen B, Yang Y (2016). A novel ammonia-oxidizing archaeon from wastewater treatment plant: its enrichment, physiological and genomic characteristics. Sci Rep.

[50] Jia ZJ, Conrad R (2009). Bacteria rather than archaea dominate microbial ammonia oxidation in an agricultural soil. Environ Microbiol.

[51] Wakelin S, Williams E, O'Sullivan CA, Cameron KC, Di HJ, Cave V, O'Callaghan M (2004). Predicting the efficacy of the nitrification inhibitor dicyandiamide in pastoral soils. Plant Soil.

[52] He Y, Hu W, Ma D, Lan H, Yang Y, Gao Y (2017). Abundance and diversity of ammonia-oxidizing archaea and bacteria in the rhizosphere soil of three plants in the Ebinur Lake wetland. Can J Microbiol.

[53] Dang H, Li J, Chen R, Wang L, Guo L, Zhang Z, Klotz MG (2010). Diversity, abundance, and spatial distribution of sediment ammonia-oxidizing Betaproteobacteria in response to environmental gradients and coastal eutrophication in Jiaozhou Bay, China. Appl Environ Microbiol.

[54] Lehtovirta-Morley LE, Ross J, Hink L, Weber EB, Gubry-Rangin C, Thion C, Prosser JI, Nicol GW (2016). Isolation of ‘Candidatus Nitrosocosmicus franklandus’, a novel ureolytic soil archaeal ammonia oxidiser with tolerance to high ammonia concentration. FEMS Microbiol Ecol.

[55] Wu YJ, Whang LM, Fukushima T, Chang SH (2013). Responses of ammonia-oxidizing archaeal and betaproteobacterial populations to wastewater salinity in a full-scale municipal wastewater treatment plant. J Biosci Bioeng.

[56] Sahan E, Muyzer G (2008). Diversity and spatio-temporal distribution of ammonia-oxidizing Archaea and Bacteria in sediments of the Westerschelde estuary. FEMS Microbiol Ecol.

[57] Hu H, Zhang LM, Yuan C, Zheng Y, Wang J, Chen D, He J (2015). The large-scale distribution of ammonia oxidizers in paddy soils is driven by soil pH, geographic distance, and climatic factors. Front Microbiol.

[58] Seo DC, Yu K, Delaune RD (2008). Influence of salinity level on sediment denitrification in a Louisiana estuary receiving diverted Mississippi River water. Arch Agron Soil Sci.

[59] Miao Y, Liao R, Zhang XX, Liu B, Li Y, Wu B, Li A (2015). Metagenomic insights into salinity effect on diversity and abundance of denitrifying bacteria and genes in an expanded granular sludge bed reactor treating high-nitrate wastewater. Chem Eng J.

[60] Francis CA, O'Mullan GD, Cornwell JC, Ward BB (2013). Transitions in nirS-type denitrifier diversity, community composition, and biogeochemical activity along the Chesapeake Bay estuary. Front Microbiol.

[61] Burgin AJ, Groffman PM. Soil O_2_ controls denitrification rates and N_2_O yield in a riparian wetland. J Geophys Res: Biogeosci. 2012;117(G1). 10.1029/2011JG001799.

[62] Philippot L, Mirleau P, Mazurier S, Siblot S, Hartmann A, Lemanceau P, Germon JC (2001). Characterization and transcriptional analysis of *Pseudomonas fluorescens* denitrifying clusters containing the nar, nir, nor and nos genes. Biochim Biophys Acta (BBA)-Gene Structure Expr.

[63] Gomes J, Khandeparker R, Bandekar M, Meena RM, Ramaiah N (2018). Quantitative analyses of denitrifying bacterial diversity from a seasonally hypoxic monsoon governed tropical coastal region. Deep-Sea Res II Top Stud Oceanogr.

[64] Zhai S, Ji M, Zhao Y, Su X (2020). Shift of bacterial community and denitrification functional genes in biofilm electrode reactor in response to high salinity. Environ Res.

[65] Mosier AC, Francis CA (2010). Denitrifier abundance and activity across the San Francisco Bay estuary. Environ Microbiol Rep.

[66] Santoro AE, Boehm AB, Francis CA (2006). Denitrifier community composition along a nitrate and salinity gradient in a coastal aquifer. Appl Environ Microbiol.

[67] Morales SE, Jha N, Saggar S (2015). Impact of urine and the application of the nitrification inhibitor DCD on microbial communities in dairy-grazed pasture soils. Soil Biol Biochem.

[68] Jones CM, Hallin S (2010). Ecological and evolutionary factors underlying global and local assembly of denitrifier communities. ISME J..

[69] Yang A, Zhang X, Agogué H, Dupuy C, Gong J (2015). Contrasting spatiotemporal patterns and environmental drivers of diversity and community structure of ammonia oxidizers, denitrifiers, and anammox bacteria in sediments of estuarine tidal flats. Ann Microbiol.

[70] Tang Y, Zhang X, Li D, Wang H, Chen F, Fu X, Yu G (2016). Impacts of nitrogen and phosphorus additions on the abundance and community structure of ammonia oxidizers and denitrifying bacteria in Chinese fir plantations. Soil Biol Biochem.

[71] Grover M, Ali SZ, Sandhya V, Rasul A, Venkateswarlu B (2011). Role of microorganismsin adaptation of agriculture crops to abiotic stresses. World J Microbiol Biotechnol.

[72] Tao R, Wakelin SA, Liang Y, Hu B, Chu G (2018). Nitrous oxide emission and denitrifier communities in drip-irrigated calcareous soil as affected by chemical and organic fertilizers. Sci Total Environ.

[73] Hu HW, Chen D, He JZ (2015). Microbial regulation of terrestrial nitrous oxide formation: understanding the biological pathways for prediction of emission rates. FEMS Microbiol Rev.

[74] Henry S, Bru D, Stres B, Hallet S, Philippot L (2006). Quantitative detection of the *nosZ* gene, encoding nitrous oxide reductase, and comparison of the abundances of 16S rRNA, *narG, nirK*, and *nosZ* genes in soils. Appl Environ Microbiol.

[75] Zhao S, Wang Q, Zhou J, Yuan D, Zhu G (2018). Linking abundance and community of microbial N_2_O-producers and N_2_O-reducers with enzymatic N_2_O production potential in a riparian zone. Sci Total Environ.

[76] Hink L, Nicol GW, Prosser JI (2017). Archaea produce lower yields of N_2_O than bacteria during aerobic ammonia oxidation in soil. Environ Microbiol.

[77] Kurola J, Salkinoja-Salonen M, Aarnio T, Hultman J, Romantschuk M (2005). Activity, diversity and population size of ammonia-oxidising bacteria in oil-contaminated landfarming soil. FEMS Microbiol Lett.

[78] Hu HW, Zhang LM, Dai Y, Di HJ, He JZ (2013). pH-dependent distribution of soil ammonia oxidizers across a large geographical scale as revealed by high-throughput pyrosequencing. J Soils Sediments.

[79] Ebie Y, Noda N, Miura H, Matsumura M, Tsuneda S, Hirata A, Inamori Y (2004). Comparative analysis of genetic diversity and expression of amoA in wastewater treatment processes. Appl Microbiol Biotechnol.

[80] Hallin S, Lindgren PE (1999). PCR detection of genes encoding nitrite reductase in denitrifying bacteria. Appl Environ Microbiol.

[81] Dong L, Meng Y, Wang J, Liu Y (2014). Evaluation of droplet digital PCR for characterizing plasmid reference material used for quantifying ammonia oxidizers and denitrifiers. Anal Bioanal Chem.

[82] Wu Y, Li Y, Fu X, Liu X, Shen J, Wang Y, Wu J (2016). Three-dimensional spatial variability in soil microorganisms of nitrification and denitrification at a row-transect scale in a tea field. Soil Biol Biochem.

